# Genomic Aromatic Compound Degradation Potential of Novel *Paraburkholderia* Species: *Paraburkholderia domus* sp. nov., *Paraburkholderia haematera* sp. nov. and *Paraburkholderia nemoris* sp. nov.

**DOI:** 10.3390/ijms22137003

**Published:** 2021-06-29

**Authors:** Sarah Vanwijnsberghe, Charlotte Peeters, Emmelie De Ridder, Charles Dumolin, Anneleen D. Wieme, Nico Boon, Peter Vandamme

**Affiliations:** 1 Laboratory of Microbiology, Department of Biochemistry and Microbiology, Ghent University, K. L. Ledeganckstraat 35, B-9000 Ghent, Belgium; Sarah.Vanwijnsberghe@ugent.be (S.V.); Charlotte.Peeters@UGent.be (C.P.); Emmelie.DeRidder@ugent.be (E.D.R.); Dumolin.charles@gmail.com (C.D.); Anneleen.Wieme@UGent.be (A.D.W.); 2 Center for Microbial Ecology and Technology, Department of Biotechnology, Faculty of Bioscience Engineering, Ghent University, B-9000 Ghent, Belgium; Nico.Boon@UGent.be

**Keywords:** *Paraburkholderia*, aromatic compound degradation, microdiversity, comparative genomics, lignin

## Abstract

We performed a taxonomic and comparative genomics analysis of 67 novel *Paraburkholderia* isolates from forest soil. Phylogenetic analysis of the *recA* gene revealed that these isolates formed a coherent lineage within the genus *Paraburkholderia* that also included *Paraburkholderia* *aspalathi*, *Paraburkholderia* *madseniana*, *Paraburkholderia* *sediminicola*, *Paraburkholderia* *caffeinilytica*, *Paraburkholderia* *solitsugae* and *Paraburkholderia* *elongata* and four unidentified soil isolates from earlier studies. A phylogenomic analysis, along with orthoANIu and digital DNA–DNA hybridization calculations revealed that they represented four different species including three novel species and *P. aspalathi*. Functional genome annotation of the strains revealed several pathways for aromatic compound degradation and the presence of mono- and dioxygenases involved in the degradation of the lignin-derived compounds ferulic acid and p-coumaric acid. This co-occurrence of multiple *Paraburkholderia* strains and species with the capacity to degrade aromatic compounds in pristine forest soil is likely caused by the abundant presence of aromatic compounds in decomposing plant litter and may highlight a diversity in micro-habitats or be indicative of synergistic relationships. We propose to classify the isolates representing novel species as *Paraburkholderia* *domus* with LMG 31832^T^ (=CECT 30334) as the type strain, *Paraburkholderia* *nemoris* with LMG 31836^T^ (=CECT 30335) as the type strain and *Paraburkholderia* *haematera* with LMG 31837^T^ (=CECT 30336) as the type strain and provide an emended description of *Paraburkholderia sediminicola* Lim et al. 2008.

## 1. Introduction

The genera *Burkholderia* and *Paraburkholderia* are nearest neighbor taxa within the class Betaproteobacteria and can be distinguished by their phylogenomic position and a trend in slightly higher genomic G+C content in the former [1]. The genus *Paraburkholderia* comprises over 70 species that appear mostly restricted to environmental samples. They are isolated from different types of soil and, occasionally, aquatic environments, plant nodules and rhizosphere samples [2,3,4,5,6,7]; yet some *Paraburkholderia* species, including *Paraburkholderia fungorum*, *Paraburkholderia tropica* and *Paraburkholderia ginsengisoli*, have been isolated from a range of infections in humans and animals [8,9,10,11,12,13].

*Paraburkholderia* species are Gram-negative rods and have a G+C content ranging from 61.4 to 65.0 mol% and a genome size between 7 and 10 Mbp. They have a wide range of metabolic capabilities, which have partly been attributed to their large genomes. Several species have nitrogen fixing abilities [7,14,15], antifungal properties [16] or can degrade aromatic compounds [17,18]. Aromatic compounds, in particular polycyclic aromatic hydrocarbons (PAHs), are important environmental contaminants in air, soil and water sources with toxic and carcinogenic properties [19,20]. The use of aromatic compound degrading bacteria for bioremediation purposes has been widely studied and several *Paraburkholderia* species appeared particularly well equipped for aromatic compound bioremediation applications [21]. Moreover, a significant part of soil organic matter consists of natural aromatic compounds in the form of lignin and tannins, amongst others. The former is one of the main polymers in plant cell walls and the most abundant natural aromatic polymer [22,23]. Furthermore, the decomposition of plant litter, a process known as humification, results in the formation of humus which contains aromatic humic substances such as humic and fulvic acid [24,25,26]. Lignocellulose is one of the main parent materials for the formation of humic substances [27,28] which represent a major component of the carbon pool in soil [29]. Lignocellulosic biomass, consisting of lignin, cellulose and hemicellulose, is also used in second generation biofuel production. However, due to the recalcitrant nature of lignin there is a high need for novel degradation methods [22,30,31]. Not only are several *Burkholderia* and *Paraburkholderia* species able to degrade xenobiotic aromatics, several strains were also reported that can degrade lignin [23,32,33,34,35].

In the course of a long-term study of the cultivable diversity of a topsoil sample of the Aelmoeseneie forest (Belgium) we isolated multiple *Paraburkholderia* strains which were closely related to *Paraburkholderia* species with well-known aromatic compound degradation capacity. In the present study, we performed a polyphasic taxonomic and comparative genomics analysis of isolates from this topsoil sample to determine the taxonomic diversity and genomic aromatic compound degradation potential of the *Paraburkholderia* species within this ecosystem. We propose to classify them within three novel *Paraburkholderia* species, each with considerable genomic potential for aromatic compound degradation.

## 2. Results

### 2.1. Isolation of Paraburkholderia Isolates

Sixty seven forest soil isolates were selected after matrix-assisted laser desorption/ionization time-of-flight mass spectrometry (MALDI-TOF MS) dereplication analyses performed in the present and previous studies [36] (Table 1). Of these isolates, 23 were picked from 1/100 diluted nutrient broth solidified with Gelzan CM (DNG), 24 from *B. cepacia* complex enrichment medium (BCEM), 17 from 1/10 diluted *Pseudomonas cepacia* azaleic acid tryptamine medium (PCAT) and 3 from modified Pikovskaya medium. Each of the 67 isolates represented a set of isolates with identical mass spectra which either remained unidentified or were identified as (*Para)burkholderia* species with low identification scores (1.70–1.99) when compared to the Bruker BDAL MSP-6309 database.

In the course of the present study, four *Burkholderia* isolates from previous studies [37,38,39,40] appeared to be closely related to isolates from the Aelmoeseneie forest soil sample (see section phylogeny). These included isolates from grassland soil, industrial coal tar-contaminated waste soil and succulent soil from the home environment of cystic fibrosis patients. An overview of all isolates is provided in Table 1.

### 2.2. Phylogeny

**Figure 1:**
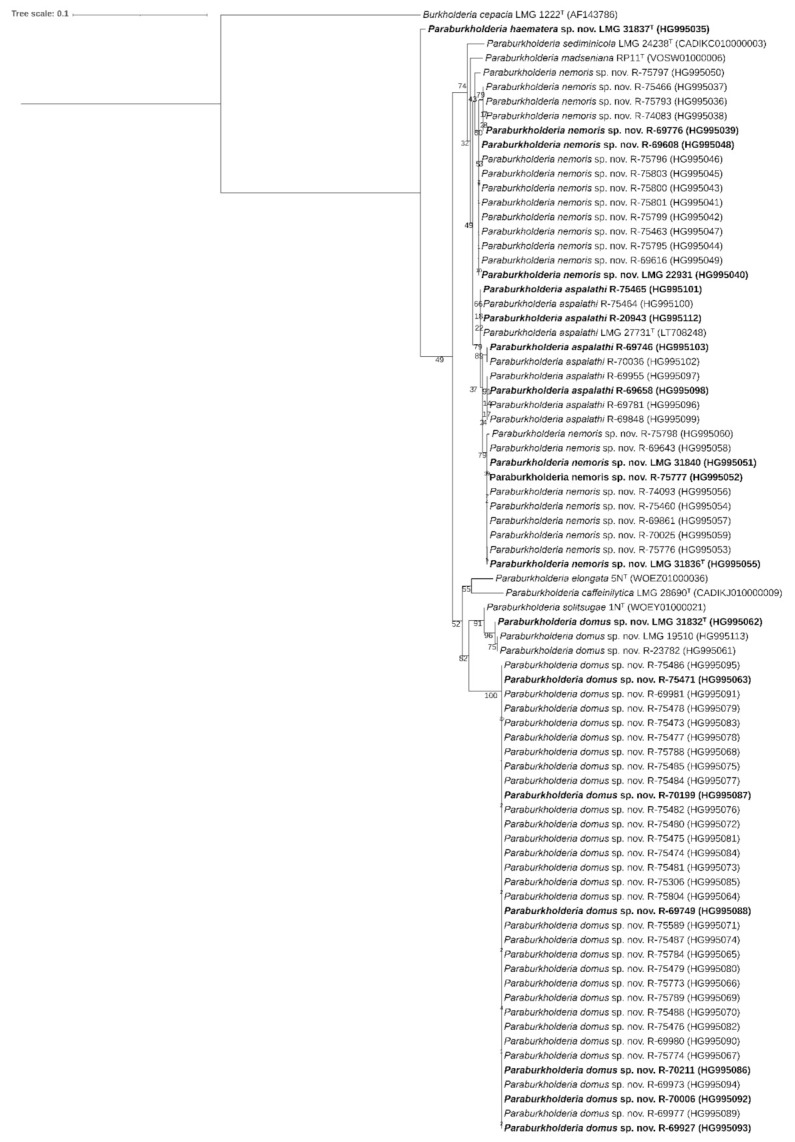
Phylogenetic tree based on *recA* gene sequences. Sequences (633–1074 bp) were aligned based on their amino acid sequences and phylogeny was inferred using the Maximum Likelihood method and GTRGAMMA substitution model in RAxML. The percentage of trees in which the associated taxa clustered together (1000 bootstrap replicates) is shown next to the branches. *Burkholderia cepacia* LMG 1222^T^ was used as outgroup. The scale bar indicates the number of substitutions per site. Isolates in bold character type were selected for whole genome sequence analysis. Sequence accession numbers are shown in parentheses.

The *recA* gene of the 67 Aelmoeseneie forest soil isolates and the four additional soil isolates was sequenced for phylogenetic analysis. In the phylogenetic tree based on the *recA* gene (Figure 1), the 71 isolates represented three clusters and a single isolate, LMG 31837^T^, that occupied a distinct position. A total of 34 isolates clustered with *P. aspalathi* LMG 27731^T^ as nearest neighbor type strain; three isolates clustered with *P. solitsugae* 1N^T^ as nearest neighbor type strain and 33 isolates formed a distinct, well-separated cluster.

**Figure 2:**
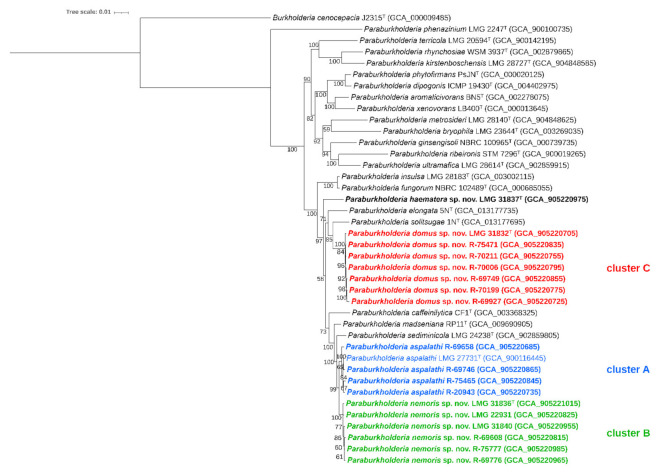
Phylogenomic tree based on 107 single-copy core genes. BcgTree was used to extract the nucleotide sequence of 107 single-copy core genes and to construct their phylogeny by partitioned maximum-likelihood analysis. The percentage of replicate trees in which the associated taxa clustered together in the bootstrap test (1000 replicates) are shown next to the branches. Eighteen isolates of the present study, the type strains of their nearest phylogenetic neighbor species and a selection of other *Paraburkholderia* type strains were included. *Burkholderia cenocepacia* J2315^T^ was used as outgroup. Strains reported in the present study are marked in bold character type. Bar, 0.01 changes per nucleotide position.

A subset of 18 representative isolates, based on their position in the *recA* gene based phylogenetic tree, was selected for whole genome sequence analysis. In addition, we also determined the whole genome sequence of *P. sediminicola* LMG 24238^T^. In a phylogenomic tree based on the analysis of 107 single-copy genes, the 18 isolates again represented a single line of descent together with the *P. aspalathi*, *P. madseniana*, *P. sediminicola*, *P. caffeinilytica*, *P. solitsugae* and *P. elongata* type strains (Figure 2). Seventeen isolates grouped in three clusters while isolate LMG 31837^T^ again occupied a well-separated position. The isolates R-20943, R-69658, R-69746 and R-75465 grouped with *P. aspalathi* LMG 27731^T^ in cluster A. Cluster B comprised LMG 31836^T^, LMG 22931, LMG 31840, R-69608, R-69776 and R-75777 and grouped with *P. aspalathi* LMG 27731^T^ as nearest neighbor type strain. Cluster C comprised isolates LMG 31832^T^, R-69749, R-69927, R-70006, R-70199, R-70211 and R-75471 and grouped with *P. solitsugae* 1N^T^ as nearest neighbor type strain.

The digital DNA-DNA hybridization (dDDH) and average nucleotide identity (orthoANIu) results generally confirmed the phylogenomic grouping as revealed in Figure 2 (Table 2 and Table 3). Cluster A isolates R-20943, R-69658, R-69746 and R-75465 yielded dDDH and orthoANIu values of at least 82.9% and 98.0%, respectively, towards *P. aspalathi* LMG 27731^T^, and maximum values of 57.4% and 94.2%, respectively, towards type strains of other *Paraburkholderia* species. The former dDDH and orthoANIu values were well above the species delineation thresholds of 70% dDDH [41] and 95–96% orthoANIu [42], and therefore cluster A isolates were assigned to *P. aspalathi*.

Cluster B isolates LMG 31836^T^, LMG 22931, LMG 31840, R-69608, R-69776 and R-75777 shared dDDH values between 89.2 and 98.0%, and orthoANIu values between 98.7 and 99.7%, again confirming they represented a single species. Average dDDH and orthoANIu values towards their closest neighbors were 62.9 ± 0.2% and 95.2 ± 0.1%, respectively, towards *P. aspalathi* LMG 27731^T^; 57.4 ± 0.2% and 94.2 ± 0.1%, respectively, towards *P. madseniana* RP11^T^; and 55.5 ± 0.1% and 93.9 ± 0.1%, respectively, towards *P. sediminicola* LMG 24238^T^. The degree of overall genomic relatedness between cluster B isolates and *P. aspalathi* LMG 27731^T^ was therefore slightly below (dDDH) or near (OrthoANIu) the species delineation thresholds. Confidence intervals of the dDDH values ranged between 59.7 and 65.9% (Supplementary Table S1), and thus remained below the species delineation threshold. Average dDDH and orthoANIu values between cluster A isolates (i.e., other *P. aspalathi* isolates) and cluster B isolates were 63.5 ± 0.8% and 95.3 ± 0.1%, i.e., slightly below (dDDH) or near (OrthoANIu) the species delineation thresholds. Again, dDDH confidence intervals ranged between 59.9 and 68.1%, and thus remained below the species delineation threshold (dDDH).

Cluster C isolates LMG 31832^T^, R-69749, R-69927, R-70006, R-70199, R-70211 and R-75471 shared dDDH values between 95.1 and 98.5%, and orthoANIu values between 99.4 and 99.8%, again confirming they represented a single species. dDDH and orthoANIu values towards type strains of other *Paraburkholderia* species were 46.5 and 91.6%, respectively, or less, and clearly far below the species delineation thresholds. Likewise, dDDH and orthoANIu values towards cluster A and B strains were well below the species delineation thresholds.

Isolate LMG 31837^T^ yielded dDDH and orthoANIu values of 44.8% and 91.2%, respectively, towards *P. elongata* 5N^T^, its nearest neighbor; 41.0% and 90.1%, respectively, towards *P. madseniana* RP11^T^; and 41.0% and 90.0%, respectively, towards *P. solitsugae* 1N^T^.

Finally, we extracted the 16S rRNA gene sequences from the draft genomes of the 18 strains. Analysis of these 16S rRNA sequences revealed *P. sediminicola* HU2-65W^T^ as the nearest neighbor taxon for all 18 strains, with sequence similarities ranging between 98.9 and 100.0%. Cluster B isolate LMG 31836^T^ exhibited sequence similarity values of 99.7% towards *P. sediminicola* HU2-65W^T^, 98.9% towards *P. madseniana* RP11^T^ and *P. aspalathi* VG1C^T^, 98.7% towards *P. insulsa* PNG-April^T^ and 98.6% towards *P. caffeinilytica* CF1^T^ and *P. kirstenboschensis* Kb15^T^. Cluster C isolate LMG 31832^T^ exhibited a sequence similarity of 98.9% towards *P. sediminicola* HU2-65W^T^ and 98.5% towards *P. metrosideri* DNBP6-1^T^. Finally, isolate LMG 31837^T^ yielded sequence similarity values of 99.2% towards *P. sediminicola* HU2-65W^T^, 98.8% towards *P. caffeinilytica* CF1^T^ and 98.7% towards *P. madseniana* RP11^T^. These high similarity values confirmed that the 16S rRNA gene lacks resolution among closely related *Paraburkholderia* species.

### 2.3. Genome Features

Genome statistics of the final genome assemblies of the 18 isolates and corresponding ENA accession numbers (PRJEB41851; *P. sediminicola* LMG 24238^T^ PRJEB37806) are presented in Table 4.

**Figure 3:**
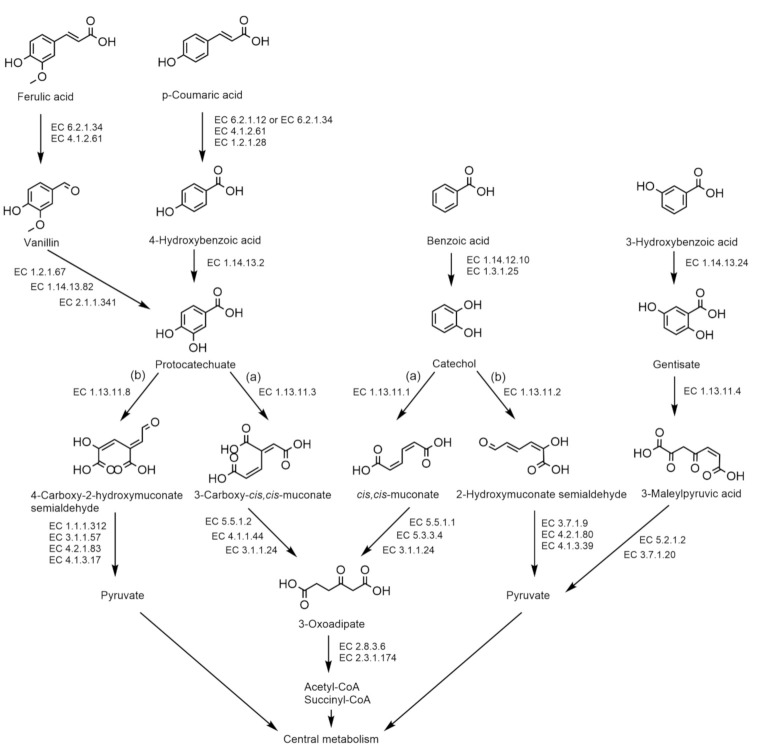
Degradation pathways of benzoic acid, 3-hydroxybenzoic acid and the lignin derived compounds ferulic acid and p-coumaric acid. (**a**) Ortho-cleavage pathway; (**b**) meta-cleavage pathway. Chemical structures were drawn with ChemDraw 19.

Functional genome annotation of the 18 selected strains and their closest neighbors *P. aspalathi* LMG 27731^T^, *P. sediminicola* LMG 24238^T^, *P. caffeinilytica* CF1^T^, *P. madseniana* RP11^T^, *P. solitsugae* 1N^T^ and *P. elongata* 5N^T^ revealed and substantiated a considerable potential for the degradation of aromatic compounds. The genomes of strains LMG 31832^T^, LMG 31836^T^ and LMG 31837^T^ contained a large number of genes in the RAST SEED subsystem category for the metabolism of aromatics, i.e., 152, 150 and 134 genes, respectively. However, the largest number of genes in this category was present in the genomes of *P. elongata* 5N^T^ (246 genes) and *P. solitsugae* 1N^T^ (217 genes). Different pathways for the degradation of aromatic compounds were encoded in the draft genomes of the examined strains (Figure 3). Pathways for which the presence of coding sequences varied within and between species are presented in Table 5. The complete pathway for aerobic catabolism of benzoic acid to catechol (EC 1.14.12.10, EC 1.3.1.25) was encoded in the genomes of all examined strains and their closest relatives. The catechol intermediate is then degraded by a catechol 1,2-dioxygenase (EC 1.13.11.1), encoded by all strains, to *cis,cis*-muconate via the ortho-cleavage pathway (Figure 3). All enzymes required for further degradation of *cis,cis*-muconate to 3-oxoadipate are encoded in the genomes of all strains examined, except LMG 31836^T^ and LMG 22931 (cluster B) and *P. aspalathi* R-20943, which lack a muconate cycloisomerase (EC 5.5.1.1) (Table 5). The enzymes required for the degradation of 3-oxoadipate (EC 2.8.3.6, EC 2.3.1.174) are encoded in the draft genomes of all examined strains. Alternatively, the catechol intermediate can be degraded to 2-hydroxymuconate semialdehyde via the meta-cleavage pathway. All cluster C strains encoded a catechol 2,3-dioxygenase (EC 1.13.11.2) for this pathway, except R-70211. LMG 31836^T^ did not encode this enzyme; however, four other cluster B strains (LMG 31840, R-69608, R-69776, R-75777) did. The enzyme was not encoded by LMG 31837^T^. Close relatives *P. elongata* 5N^T^ and *P. aspalathi* R-20943 also encoded a catechol 2,3-dioxygenase. Genes for complete further degradation of 2-hydroxymuconate semialdehyde were only present in one cluster C strain, R-69927. Cluster B strain R-69608 encoded a 2-oxopent-4-enoate hydratase (EC 4.2.1.80), which was also present in all cluster C strains but not in other cluster B strains. Cluster B strains LMG 22931 and LMG 31840 did not encode a 4-hydroxy-2-oxovalerate aldolase (EC 4.1.3.39), which was present in all cluster C strains, LMG 31837^T^ and other cluster B strains. The genomes of all strains encoded a benzoate-CoA ligase (EC 6.2.1.25), a key enzyme for the conversion of benzoate to benzoyl-CoA. However, all strains lacked genes encoding essential enzymes for further anaerobic degradation of benzoyl-CoA. The isolates LMG 31832^T^, LMG 31836^T^ and LMG 31837^T^ did not encode a 3-hydroxybenzoate 6-monooxygenase (EC 1.14.13.24), nor did any other cluster B or C isolate. This enzyme is present in *P. elongata* 5N^T^ and is a key enzyme for the degradation of 3-hydroxybenzoic acid to gentisate. None of the strains of the newly described species encoded a gentisate 1,2-dioxygenase (EC 1.13.11.4) for ring cleavage of the gentisate intermediate (Figure 3). Close relatives *P. aspalathi* R-20943, R-69746 and R- 75465, *P. madseniana* RP11^T^, *P. solitsugae* 1N^T^ and *P. elongata* 5N^T^ encoded a gentisate 1,2-dioxygenase. All strains encoded the essential enzymes for further degradation of 3-maleylpyruvic acid.

The ability to degrade aromatic compounds can be attributed to the presence of natural aromatic polymers in the soil in the form of, amongst others, lignin, tannins and humic substances. While the present literature has mainly focused on the capacity of *Burkholderia* and *Paraburkholderia* strains to degrade xenobiotic aromatic compounds, they have also been reported as bacteria that can degrade natural aromatic polymers such as lignin [23,32,33,34]. The presence of genes encoding DyP-type peroxidases, catalase-peroxidases and superoxide dismutases in the genomes of all strains suggests the ability to depolymerize lignin into oligomeric, dimeric and monomeric lignin-derived compounds (LDCs) [43]. The draft genomes of all strains encoded several enzymes involved in the degradation of monomeric LDCs such as ferulic acid and p-coumaric acid. Figure 3 shows the pathways through which these LDCs can be degraded. The enzymes required for the degradation of the LDCs and their degradation intermediates are presented in Table 5 (for coding sequences that varied within and between species). Ferulic acid is converted to vanillin via CoA ligation by feruloyl-CoA synthase (EC 6.2.1.34), which is then further degraded to protocatechuate. The complete pathway for conversion to vanillin is encoded in the draft genomes of all strains except *P. caffeinilytica* CF1^T^. However, none of the strains encoded the complete pathway for further degradation to protocatechuate. p-Coumaric acid is also converted by CoA ligation to 4-hydroxybenzoic acid and then further degraded to protocatechuate. All strains lacked the 4-coumarate-CoA ligase enzyme (EC 6.2.1.12) for CoA ligation, however, p-coumaric acid can also be converted by feruloyl-CoA synthase due to its broad substrate range [44]. The necessary enzymes for further degradation to 4-hydroxybenzoic acid and protocatechuate are present in the genomes of all strains, with the exception of a benzaldehyde dehydrogenase (EC 1.2.1.28) in all cluster C strains, including LMG 31832^T^. The protocatechuate intermediate is then further degraded to pyruvate via the meta-cleavage pathway or to acetyl-CoA and succinyl-CoA via the ortho-cleavage pathway. All examined strains encoded the complete ortho-cleavage pathway of protocatechuate via 3-oxoadipate (EC 1.13.11.3, EC 5.5.1.2, EC 4.1.1.44, EC 3.1.1.24, EC 2.8.3.6, EC 2.3.1.174). All strains, except *P. caffeinilytica* CF1^T^, encoded a protocatechuate 4,5-dioxygenase (EC 1.13.11.8) for ring cleavage via the meta-cleavage pathway. All cluster C strains, one cluster B strain (LMG 22931) and *P. solitsugae* 1N^T^ encoded all necessary enzymes for further degradation of 4-carboxy-2-hydroxymuconate semialdehyde to pyruvate. Two cluster B strains (R-69776 and R-75777) lacked the presence of a 2-hydroxy-4-carboxymuconate 6-semialdehyde dehydrogenase (EC 1.1.1.312) but encoded all other enzymes for degradation to pyruvate.

### 2.4. Physiology

A phenotypic characterization of isolates LMG 31832^T^, LMG 31836^T^, LMG 31837^T^ and their closest neighbors, *P. aspalathi* LMG 27731^T^, *P. sediminicola* LMG 24238^T^, *P. caffeinilytica* CF1^T^, *P. madseniana* RP11^T^, *P. solitsugae* 1N^T^ and *P. elongata* 5N^T^ was performed.

Cells of the examined strains were rod-shaped, non-motile and occurred alone or in pairs. Cells of strains LMG 31832^T^ and LMG 31836^T^ were approximately 2 µm long, cells of strain LMG 31837^T^ were about 1 µm long. All cells were about 0.5 µm in diameter. After three days of incubation on NAB at 28 °C, colonies were circular with an entire edge and a smooth surface. They were flat or slightly raised, beige and opaque. They had a diameter of 1 mm.

After three days of incubation, all strains shared the following characteristics: growth on TSA and NAB at 20 and 28 °C, growth on MacConkey agar, on TSA supplemented with horse blood, growth at pH 6 and 7 and growth on Tween 20, 40, 60 and 80. The following characteristics were uniformly observed after seven days of incubation: growth on TSA and NAB at 15 °C and growth on Drigalski agar. When tested with the API 20NE microtest system, the following characteristics were present after 48 h of incubation: esculin hydrolysis and assimilation of glucose, mannose, mannitol, N-acetyl-glucosamine, potassium gluconate and malate. The following enzyme activities of the API ZYM microtest were present: butyrate esterase (C4) (weakly), caprylate esterase lipase (C8) (weakly), leucyl arylamidase and acid phosphatase.

The following characteristics were uniformly absent: growth on TSA and NAB at 4, 37, 40 and 45 °C, growth on cetrimide agar, growth in the presence of 2% to 10% of NaCl and at pH 4, 5, 8 and 9. No growth was observed on NAB, TSA and TSA supplemented with 10 mM KNO_3_ under anaerobic atmosphere. Hydrolysis of starch and casein and DNase activity were absent. The following characteristics were absent when examined with the API 20NE microtest system: indol production from L-tryptophane, fermentation of glucose, arginine dihydrolase and urease activity, gelatin liquefaction and assimilation of capric acid. The following characteristics were absent when examined with the API ZYM microtest system: myristate lipase (C14), valine arylamidase, cystin arylamidase, trypsin, chymotrypsin, alpha-galactosidase, beta-galactosidase, beta-glucuronidase, alpha-glucosidase, beta-glucosidase, N-acetyl-beta-glucosaminidase, alpha-mannosidase and alpha-fucosidase.

Strains LMG 31832^T^, LMG 31836^T^, LMG 31837^T^ and their closest phylogenetic neighbors could be differentiated based on several characteristics (Table 6). The lack of beta-galactosidase activity distinguished strains LMG 31832^T^, LMG 31836^T^ and LMG 31837^T^ from *P. aspalathi* LMG 27731^T^ and *P. sediminicola* LMG 24238^T^. Strains LMG 31832^T^ and LMG 31836^T^ were also differentiated from their closest neighbors by their ability to reduce nitrate and could be differentiated from each other based on assimilation of N-acetyl-glucosamine, trisodium citrate and phenylacetic acid. Strain LMG 31837^T^ could be differentiated from its closest neighbor *P. elongata* 5N^T^ based on hemolysis on horse blood agar and growth in 1% NaCl.

### 2.5. MALDI-TOF MS Analysis

**Figure 4:**
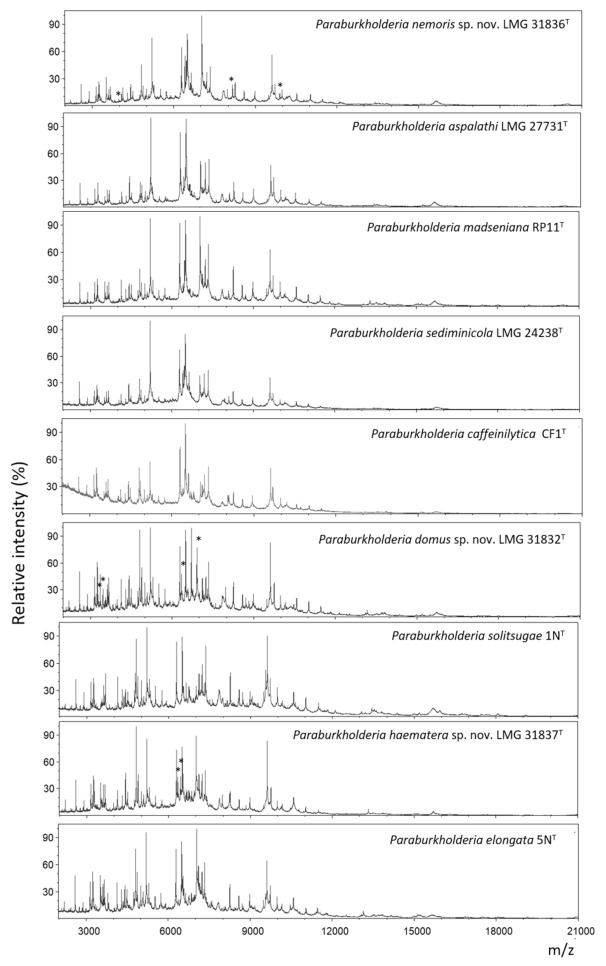
MALDI-TOF MS profiles of strains LMG 31832^T^, LMG 31836^T^, LMG 31837^T^ and their closest phylogenetic neighbor type strains, ordered conform to genomic relatedness. Asterisks indicate peaks that allow differentiation of the strain from its closest neighbors.

All strains had highly similar MALDI-TOF MS profiles (Figure 4). Strain LMG 31836^T^ showed two peaks at *m/z* 8152.8 ± 0.5 and 9892.3 ± 0.2 that were not present in the spectra of its closest neighbors *P. aspalathi* LMG 27731^T^, *P. madseniana* RP11^T^ and *P. sediminicola* LMG 24238^T^. Strain LMG 31836^T^ could also be differentiated from *P. madseniana* RP11^T^ and *P. sediminicola* LMG 24238^T^ by a peak at 4076.2 ± 0.4. Strain LMG 31832^T^ could be differentiated from its closest neighbor *P. solitsugae* 1N^T^ by four peaks at *m/z* 3350.4 ± 0.5, 3451.4 ± 0.7, 6336.8 ± 0.7 and 6905.4 ± 0.8. Moreover, *P. solitsugae* 1N^T^ showed two peaks at *m/z* 6634.2 ± 1.8 and 7006.5 ± 2.2 that were not present in the spectra of strain LMG 31832^T^. Finally, LMG 31837^T^ could be differentiated from its closest neighbor *P. elongata* 5N^T^ by two peaks at *m/z* 6325.5 ± 1.0 and 6425.9 ± 0.7.

## 3. Discussion

In the frame of several efforts to maximally isolate the cultivable bacterial community of a topsoil sample of the Aelmoeseneie forest, more than 5000 isolates have been examined thus far ([36] and data from the present study). These isolates have all been dereplicated by MALDI-TOF MS using the SPeDE algorithm [45] to remove redundant isolates. The resulting SPeDE ‘references’ are strains with distinct MALDI-TOF mass spectra, as a proxy to isolates with a distinct genetic background [45] and included 67 isolates examined in the present study. *RecA* gene sequence analysis identified four additional isolates from earlier studies [37,38,39,40] that were closely related to the 67 forest soil isolates.

We used *recA* gene sequencing to subsequently select 18 isolates for comparative genomics analyses, in order to represent the observed diversity in *recA* gene sequences in a redundant manner (Figure 1). Their position in the *recA* and phylogenomic trees (Figure 1; Figure 2) and the dDDH and orthoANIu values (Table 2; Table 3) allowed to assign nine out of 71 isolates to *P. aspalathi*, 25 to the taxon represented by strain LMG 31836^T^, 36 to the taxon represented by strain LMG 31832^T^ and a single isolate to the taxon represented by LMG 31837^T^. The divergence in *recA* gene sequences and the dDDH and OrthoANIu values among isolates for which draft genome sequences were generated demonstrated considerable genetic divergence within species for which multiple isolates were studied. Data from the present study therefore demonstrated that the 67 isolates from the Aelmoeseneie forest soil sample and four soil isolates from earlier studies [37,38,39,40] represented multiple clonal variants within four genospecies, one of which was identified as *P. aspalathi*, an organism from root nodules of a South African legume plant [5]. This included strain R-20943 (=Hg8) which was isolated from a coal tar-contaminated hillside soil in the USA [37].

A second group of 25 isolates, represented by strain LMG 31836^T^, appeared to be closely related *to P. aspalathi* LMG 27731^T^ with average dDDH values of 62.9 ± 0.2% and average orthoANIu values of 95.2 ± 0.1% (Table 2; Table 3). The dDDH value was slightly below the species delineation threshold of 70% and was supported by confidence intervals ranging between 59.7% and 65.9%. In contrast, the orthoANIu value was at the species delineation threshold. Strain LMG 31836^T^ could be differentiated from *P. aspalathi* LMG 27731^T^ by several phenotypic characteristics (Table 6) and by its MALDI-TOF MS profile (Figure 4) which argued against a classification as *P. aspalathi*. Classifying these 25 isolates into a novel subspecies within *P. aspalathi* would not be appropriate either as subspecies typically share dDDH levels of 80% or higher [46]. Together, these data suggest that overall genomic relatedness values that are near the species delineation thresholds should be used in concert with other data to propose a classification that best reflects all data. We consider it most appropriate to classify strain LMG 31836^T^ into a novel *Paraburkholderia* species. Isolates of this novel species have genomes with an average G+C content of 61.4 ± 0.1 mol% and which range in size between 8.64 and 9.28 Mbp (Table 4). This novel *Paraburkholderia* species and *P. aspalathi* cannot be distinguished through *recA* gene sequence analysis which corroborates the close genomic relatedness of both taxa (Figure 1). Strain LMG 22931 (=G47-12) was isolated from grassland soil in the Netherlands in 2003 and belongs to the same novel *Paraburkholderia* species [40].

A third group of 36 isolates, represented by strain LMG 31832^T^, formed a well separated novel species within this *Paraburkholderia* lineage with *P. solitsugae* 1N^T^ as its nearest neighbor taxon (Figure 2) and could be distinguished from the type strains of its nearest neighbor taxa through various genomic and phenotypic analyses (Figure 1; Figure 2, Table 2, Table 3 and Table 6). Isolates of this novel species have genomes with an average G+C content of 61.3 ± 0.1 mol% and which range in size between 8.19 and 9.46 Mbp (Table 4). This novel species also includes strain LMG 19510 (=DSM 6431) which was isolated from industrial waste deposit soil in Germany [38] and strain R-23782 which was isolated from soil of a succulent plant in the home of a cystic fibrosis patient in Belgium [39].

Finally, strain LMG 31837^T^ also represented a well-separated novel species within this *Paraburkholderia* lineage with *P. elongata* 5N^T^ as its nearest neighbor taxon (Figure 2) and could be distinguished from the type strains of its nearest neighbor taxa through various genomic and phenotypic analyses (Figure 1; Figure 2, Table 2, Table 3 and Table 6). Its genome has a G+C content of 61.71 mol% and is 7.82 Mbp in size (Table 4). Remarkably, among the more than 5000 isolates that were examined in the course of several isolation campaigns, this was the only isolate that represented this novel species.

Like several of their nearest neighbor taxa and other *Paraburkholderia* species [2,18,21] these novel species have a metabolism that is highly adapted to the degradation of aromatic compounds. While the present literature has mainly focused on the capacity of *Burkholderia* and *Paraburkholderia* strains to degrade xenobiotic aromatic compounds, they have also been reported as bacteria that can degrade lignin [23,32,33,34] which is the most abundant natural aromatic polymer [23,47]. We hypothesize that the co-occurrence of multiple closely related *Paraburkholderia* strains and species with the capacity to degrade aromatic compounds in pristine forest soil is related to the presence of structurally complex, natural aromatic compounds such as lignin, tannins and humic substances generated through the decomposition of plant litter [24,25,26]. The degradation of such aromatic polymers, in particular lignin, has mostly been described for fungi [48]. Nonetheless, the genomes of all strains examined in the present study encoded enzymes involved in the depolymerization of lignin and the degradation of monomeric LDCs. A mutualistic relationship may exist between fungi and bacteria in soil, in which bacteria consume lignin degradation products generated by fungi [49,50]. Mutualistic relations between fungi and *Burkholderia* (now *Paraburkholderia*) in soils have indeed been described before [51]. The co-occurrence of multiple closely related *Paraburkholderia* strains and species in a single topsoil sample may highlight a diversity in micro-habitats with different species and strains occupying different micro-niches [52], or may be indicative of synergistic relationships, whereby strains encode different parts of degradation pathways. The latter is supported by the fact that the genomes of the examined species and strains did not encode the exact same pathways for aromatic compound degradation.

The observation that genetically distinct but closely related strains partition their environment at fine phylogenetic and phylogenomic scales is a finding that is not well understood. The existence and function of such microdiversity, i.e., the co-occurrence of closely related but ecologically and physiologically distinct taxonomic groups has been documented for about two decades and is an intrinsic property of many microorganisms [53]. Among others, microdiversity has been attributed to resource use and phosphate acquisition [52]. A deeper mining of the genomic and phenotypic potential of different strains of each of these four *Paraburkholderia* species will be required to improve our understanding of the functional role of this microdiverse *Paraburkholderia* community.

## 4. Materials and Methods

### 4.1. Isolation of Paraburkholderia Isolates

The Aelmoeseneie forest (Gontrode, Belgium: 50°58′ N 3°49′ E) is a temperate deciduous forest of 28 ha with mixed tree species (oak, beech, ash and others) and a lush undergrowth located in northwestern Belgium [54]. The southeastern part of the forest is considered ancient as it has been continuously forested since at least the oldest land-use map of 1775 [55,56]. One square meter of the top layer of soil (0–10 cm depth) was collected, sieved through a sterile brass sieve (aperture 1 cm), homogenized using a new concrete mixer that was decontaminated using sodium hypochlorite, aliquoted into 250 mL Nalgene© bottles and stored at −80 °C. In the frame of several studies, we performed large-scale campaigns to isolate the cultivable bacterial community of this soil sample.

For isolation experiments, 9 g of soil was mixed with 90 mL sodium pyrophosphate (0.05 M, pH 8) for 3 min. To maximize harvesting all cells, this suspension was centrifuged 4 times for 5 min. at 130× *g*, 2 times for 5 min. at 60× *g*, 2 times for 1 min. at 40× *g* and finally 2 times for 1 min. at 20× *g*. Between each centrifugation step, the supernatants were transferred and the pellet was resuspended in 45 mL sodium pyrophosphate and mixed for 3 min. Subsequently, all cell fractions were centrifuged for 15 min. at 8226× *g*, the supernatants were removed, and the cell pellets were resuspended in 5 mL saline (0.85% NaCl). Finally, all cell fractions were pooled and a 10-fold dilution series was prepared in saline and plated (i) onto 1/100 diluted nutrient broth (Oxoid, Basingstoke, UK) solidified with Gelzan CM (1 g L^−1^;Sigma-Aldrich, St.-Louis, MO, USA) (DNG) and supplemented with MgSO_4_·7H_2_O (1 g L^−1^) and cycloheximide (1 g L^−1^) and (ii) onto 1/10 diluted *Pseudomonas cepacia* azaleic acid tryptamine (PCAT) medium [57]. Isolates from DNG were picked after 4 weeks of incubation at 20 °C and transferred to 96-well microtiter plates containing 1/10 diluted nutrient broth (DNB; Oxoid, Basingstoke, UK). Isolates from 1/10 PCAT were picked after 2 weeks of incubation at 20 °C and transferred to 96-well microtiter plates containing tryptone soy broth (TSB; Oxoid, Basingstoke, UK). Isolates were subcultivated using the VIAFLO 96/384 liquid handler (Integra-Biosciences©, Zizers, Switzerland) until the third generation and cell suspensions were prepared for MALDI-TOF MS as described earlier [45].

Additionally, 9 g of soil sample was homogenized in 90 mL physiological saline for 3 min. A 10-fold serial dilution in saline was prepared and plated on *B. cepacia* complex enrichment medium (BCEM) [57] and on modified Pikovskaya medium for the isolation of phosphate solubilizers [58]. After 5 days of incubation at 20 °C isolates were picked and subcultivated until the third generation on phosphate buffered (0.45 g L^−1^ KH_2_PO_4_/2.39 g L^−1^ Na_2_HPO_4_·12H2O) nutrient agar (NAB; Oxoid, Basingstoke, UK) or modified Pikovskaya medium, respectively. Approximately 1 µL loop of biological material of the isolates picked from BCEM was harvested and transferred to sterile deepwell plates (Thermo Scientific, Roskilde, Denmark) containing phosphate buffered nutrient broth (BNB; Oxoid, Basingstoke, UK). After an incubation period of 6 days at 20 °C, cell suspensions for MALDI-TOF MS analysis were prepared as described earlier [45]. For the isolates picked from Pikovskaya medium, extracts were prepared for MALDI-TOF MS analysis as described previously [59].

MALDI-TOF MS spectra were generated for all isolates using a Bruker Microflex^TM^ LT/SH system (Bruker Daltonics, Bremen, Germany). Spectra were dereplicated with the SPeDE algorithm [45].

### 4.2. Phylogeny

The *recA* gene of 67 forest soil isolates and four additional soil isolates was sequenced for phylogenetic analysis. DNA was extracted by adding 1 µL loop of cell material to 20 µL of alkaline lysis buffer containing 0.25% (*w*/*v*) SDS and 0.05 M NaOH and heating to 95 °C for 15 min. After lysis, 180 µL of distilled water was added. The *recA* gene (663 bp) was amplified using a Taq DNA polymerase kit (Qiagen, Antwerp, Belgium) with forward primer 5′-AGGACGATTCATGGAAGAWAGC-3′ and reverse primer 5′- GACGCACYGAYGMRTAGAACTT-3′ [60,61]. The resulting products were purified using a NucleoFast 96 PCR clean-up kit (Macherey-Nagel, Eupen, Belgium). Purified products were sequenced by a commercial company (Eurofins, Ebersberg, Germany) using the above-described primers, a 3730xl Genetic Analyzer (Thermo Fisher Scientific, Roskilde, Denmark) and the Big Dye terminator v3.1 cycle sequencing kit (Thermo Fisher Scientific, Roskilde, Denmark). Sequences were assembled using BioNumerics version 7.6 (Applied Maths, Sint-Martens-Latem, Belgium). All *recA* gene sequences were compared with those of the type strains of all validly named *Paraburkholderia* species (data not shown). Sequences (633–1074 bp) were aligned based on amino acid sequences using Muscle [62] in MEGA X [63]. A phylogenetic tree of the 71 isolates and of the type strains of their nearest neighbor taxa, i.e., *P. aspalathi* LMG 27731^T^, *P. madseniana* RP11^T^, *P. sediminicola* LMG 24238^T^, *P. caffeinilytica* CF1^T^, *P. solitsugae* 1N^T^ and *P. elongata* 5N^T^, was constructed using RAxML version 8.2.12 [64] with the GTRGAMMA substitution model and 1000 bootstrap analyses. All *recA* gene sequences were submitted into the European Nucleotide Archive (ENA) and accession numbers can be found in Figure 1.

Using their position in the *recA* gene based phylogenetic tree (Figure 1) a subset of 18 representative isolates was subsequently selected for whole genome sequence analysis to assess the genomic divergence within and between each of the clusters and the *Paraburkholderia* type strains. In addition, we determined the whole genome sequence of *P. sediminicola* LMG 24238^T^.

DNA was extracted using an automated Maxwell^®^ DNA preparation instrument (Promega, Madison, WI, USA). DNA extracts were treated with RNAse (2 mg mL^−1^, 5 µL per 100 µL extract) and incubated at 37 °C for one hour. DNA quality was checked using 1% agarose gel electrophoresis and DNA quantification was performed using the QuantiFluor ONE dsDNA system and the Quantus fluorometer (Promega, Madison, WI, USA). In the first series of analyses, draft genomes of strains R-69608, R-69658, R-69746, R-69749, R-69776, R-69927, R-70006, R-70199, R-70211 and LMG 31837^T^ were sequenced using the Illumina HiSeq4000 (PE150) platform at the Oxford Genomics Centre (Oxford, UK). In the second series of analyses, draft genomes of strains R-20943, R-75465, R-75777, R-75471, LMG 22931, LMG 31832^T^, LMG 31836^T^ and LMG 24238^T^ were sequenced using the Illumina NovaSeq6000 (PE150) platform. Finally, the draft genome of LMG 31840 was sequenced at the MiGS center (Pittsburgh, USA) using the Illumina NextSeq550 (PE150) platform. Quality reports were created with FastQC version 0.11.8. Prior to assembly, reads were trimmed (Phred score >Q30) and filtered (length >50 bp) with fastp 0.20.0 [65] with the correction option enabled. Assembly was performed with Shovill version 1.1.0, with SPAdes genome assembler 3.14.0 at its core and read error correction disabled. Contigs shorter than 500 bp were removed from the final assembly. Quality of the final assembly was verified with The Quality Assessment Tool for Genome Analysis (QUAST) which generates summary statistics such as the number of contigs, N50, L50 and the G+C content [66]. Raw reads were mapped against the assemblies using BWA-MEM [67]. Mapped reads were subjected to a quality control using Qualimap [68] to determine the coverage and error rate. Finally, the assemblies were checked for completeness and contamination using CheckM version 1.1.2 [69]. Annotation was performed using Prokka 1.14.5 [70]. The annotated genome assemblies were submitted into ENA and are publicly available under PRJEB41851 and PRJEB37806.

The genomes were submitted to the Type (Strain) Genome Server (TYGS) (accessed on 26-01-2021) [71] to identify their nearest phylogenomic neighbors and to calculate their degree of relatedness towards these nearest neighbor species. Digital DNA-DNA hybridization (dDDH) values and confidence intervals were calculated using the recommended settings of the GGDC 2.1. [41]. The average nucleotide identity (ANI) values were calculated using the OrthoANIu algorithm [72]. The whole-genome sequences of the 18 isolates and of the type strains of closely related *Paraburkholderia* species (downloaded from GenBank except for *P. sediminicola* LMG 24238^T^) were also used to construct a phylogenomic tree based on the analysis of 107 single-copy core genes using bcgTree [73] with *Burkholderia cenocepacia* J2315^T^ as outgroup. Visualization and annotation of the tree was performed using iTOL [74].

For completeness, 16S rRNA gene sequences were extracted from the draft genomes of all 18 isolates using the BAsic Rapid Ribosomal RNA Predictor software (Barrnap) [75] and were submitted to the EZBiocloud identification service (accessed on 25-02-2020) [76]. The 16S rRNA gene sequences of the type strains LMG 31832^T^, LMG 31836^T^ and LMG 31837^T^ were submitted into ENA and are publicly available under HG995123, HG995124 and HG995125, respectively.

### 4.3. Functional Genome Annotation

The assembled genomes of the 18 selected isolates and of *P. aspalathi* LMG 27731^T^, *P. sediminicola* LMG 24238^T^, *P. caffeinilytica* CF1^T^, *P. madseniana* RP11^T^, *P. solitsugae* 1N^T^ and *P. elongata* 5N^T^ were submitted to RAST (accessed on 17-12-2020) [77] and eggNOG-mapper v2 (version 2.1.4) (accessed on 23-06-2021) [78,79] for functional genome annotation with a focus on aromatic compound degradation, a key characteristics of *P. madseniana*, *P. solitsugae* and *P. elongata* [2,18].

### 4.4. Physiology

A phenotypic characterization of isolates LMG 31832^T^, LMG 31836^T^, LMG 31837^T^ and their closest neighbors, *P. aspalathi* LMG 27731^T^, *P. sediminicola* LMG 24238^T^, *P. caffeinilytica* CF1^T^, *P. madseniana* RP11^T^, *P. solitsugae* 1N^T^ and *P. elongata* 5N^T^ was performed.

Cell and colony morphology were assessed after three days of incubation on phosphate buffered (0.45 g/L KH_2_PO_4_/2.39 g/L Na_2_HPO_4_·12H_2_O) nutrient agar (NAB; Oxoid, Basingstoke, UK) at 28 °C. Motility was observed in young cultures by examining wet mounts in broth by phase-contrast microscopy. The assessment of catalase and oxidase activities and other tests were performed using conventional procedures [80]. Aerobic growth was tested at 28 °C on NAB, TSA, MacConkey agar (Oxoid, Basingstoke, UK), Drigalski agar (Biorad, Temse, Belgium) and *Pseudomonas* cetrimide agar (Oxoid, Basingstoke, UK). The temperature growth range was tested on NAB and TSA at 4, 15, 20, 28, 37, 40 and 45 °C. The effect of NaCl on growth was investigated in nutrient broth (Oxoid, Basingstoke, UK) with different concentrations of NaCl (0–10% with 1% intervals, *w*/*v*). The pH range for growth was evaluated in nutrient broth buffered at pH 4.0 to 9.0 at intervals of 1 pH unit using the following buffer systems: acetate buffer (4.0–5.0), phosphate buffer (pH 6.0–8.0) and Tris-HCl (pH 9.0). Anaerobic growth was tested on NAB, TSA and TSA supplemented with 10 mM KNO_3_. Growth tests were read after 1, 2, 3, 5 and 7 days of incubation. Hemolysis of horse blood was tested on TSA supplemented with 5% horse blood. DNase activity was tested using DNase agar (Sigma-Aldrich, St.-Louis, MO, USA). Starch hydrolysis was tested using NAB and TSA supplemented with 0.8% (*w*/*v*) soluble starch. Hydrolysis of casein was tested using Plate count agar (Oxoid, Basingstoke, UK) supplemented with 1.3% (*w*/*v*) dried skim milk (Oxoid, Basingstoke, UK). Hydrolysis of Tween 20, 40, 60 and 80 was tested as described by Sierra [81]. The activity of constitutive enzymes and other physiological properties were determined after growth on NAB for 2 days at 28 °C using the API 20NE and API ZYM microtest systems (BioMérieux, Marcy l’Etoile, France), according to the manufacturer’s instructions. Results were read after 24 h and 48 h, and after 4 h of incubation, respectively.

### 4.5. MALDI-TOF MS Analysis

MALDI-TOF MS profiles of strains LMG 31832^T^, LMG 31836^T^, LMG 31837^T^ and their closest neighbors were obtained as described before [45]. The strains were cultivated on NAB at 28 °C for 2 days before cell extracts were prepared. Two technical and two biological replicates were obtained for each strain. The profiles were visualized using mMass 5.5.0 [82].

## 5. Conclusions

In conclusion, data from the present study confirmed that strains LMG 31832^T^, LMG 31836^T^ and LMG 31837^T^ represent three novel *Paraburkholderia* species, which can be distinguished from their nearest neighbor species using genomic (Table 2; Table 3, Figure 1; Figure 2), phenotypic (Table 6) and chemotaxonomic (Figure 4) characteristics.

We propose to classify the isolates examined in the present study into three novel *Paraburkholderia* species as *Paraburkholderia domus* sp. nov. with LMG 31832^T^ (=CECT 30334) as the type strain, *Paraburkholderia nemoris* sp. nov. with LMG 31836^T^ (=CECT 30335) as the type strain and *Paraburkholderia haematera* sp. nov. with LMG 31837^T^ (=CECT 30336) as the type strain.

### 5.1. Description of Paraburkholderia domus sp. nov.

*Paraburkholderia domus* sp. nov. (do’mus. L. fem. gen. n. *domus*, from a home, household; because one of the earliest isolates of this species was collected from the home environment of a person with cystic fibrosis).

Cells are non-motile, rod-shaped, approximately 2 µm long and 0.5 µm wide. After incubation on NAB at 28 °C for three days, colonies have a diameter of 1 mm and are circular, opaque and beige. They are flat or slightly raised and have an entire edge and a smooth surface. Strain LMG 31832^T^ optimally grows on NAB and TSA between 15 and 28 °C in aerobic conditions, and does not grow in anaerobic conditions on NAB, TSA or TSA supplemented with 10 mM KNO_3_. No growth at 4, 37, 40 and 45 °C. Growth is observed in the presence of 0% and 1% NaCl, but not 2% or more. Growth at pH 6.0 and 7.0, but not at 4.0, 5.0, 8.0 or 9.0. Growth on MacConkey and Drigalski agar, no growth on cetrimide agar. Growth on Tween 20, 40, 60 and 80 agar base and hydrolysis of all Tweens after 7 days of incubation. Hemolysis on horse blood agar but no DNase activity and hydrolysis of starch or casein. Oxidase and weak catalase activity is present.

When examined with the API 20NE microtest system, the following characteristics are present: nitrate reduction, esculin hydrolysis, assimilation of glucose, arabinose (weakly), mannose, mannitol, potassium gluconate, adipic acid and malate (weakly) but not indol production from L-tryptophane, fermentation of glucose, arginine dihydrolase, urease and beta-galactosidase (PNPG) activity, gelatin liquefaction and assimilation of N-acetyl-glucosamine, maltose, capric acid, trisodium citrate and phenylacetic acid.

When examined with the API ZYM microtest system, the following characteristics are present: alkaline phosphatase (weakly), butyrate esterase (C4) (weakly), caprylate esterase lipase (C8) (weakly), leucyl arylamidase, acid phosphatase and phosphoamidase but not myristate lipase (C14), valine arylamidase, cystin arylamidase, trypsin, chymotrypsin, alpha-galactosidase, beta-galactosidase, beta-glucuronidase, alpha-glucosidase, beta-glucosidase, N-acetyl-beta-glucosaminidase, alpha-mannosidase and alpha-fucosidase.

The type strain, LMG 31832^T^ (=CECT 30334), was isolated in 2018 from forest soil. Other strains were isolated from soil of a succulent plant in the home environment of a person with cystic fibrosis and from industrial waste deposit soil (Table 1). The whole-genome sequence of strain LMG 31832^T^ has a size of 8.75 Mbp. The G+C content is 61.32 mol%. The 16S rRNA gene, *recA* gene and whole-genome sequences of strain LMG 31832^T^ are publicly available under accession numbers HG995123, HG995062 and CAJNAT000000000, respectively.

### 5.2. Description of Paraburkholderia nemoris sp. nov.

*Paraburkholderia nemoris* sp. nov. (ne.mo’ris. L. neut. gen. n. *nemoris*, from a forest; because most isolates of this species were obtained from a forest soil sample).

Cells are non-motile, rod-shaped, approximately 2 µm long and 0.5 µm wide. After incubation on NAB at 28 °C for three days, colonies have a diameter of 1 mm and are circular, opaque and beige. They are flat or slightly raised and have an entire edge and a smooth surface. Strain LMG 31836^T^ grows optimally on NAB and TSA between 15 and 28 °C in aerobic conditions, but does not grow in anaerobic conditions on NAB, TSA or TSA supplemented with 10 mM KNO_3_. No growth at 4, 37, 40 and 45 °C. Growth is observed in the presence of 0 and 1% NaCl, but not 2% or more. Growth at pH 6.0 and 7.0, but not at 4.0, 5.0, 8.0 or 9.0. Growth on MacConkey agar, delayed growth on Drigalski agar and no growth on cetrimide agar. Growth on Tween 20, 40, 60 and 80 agar base and hydrolysis of all Tweens after 7 days of incubation. Hemolysis on horse blood agar but no DNase activity and hydrolysis of starch or casein. Oxidase and weak delayed catalase activity is present.

When examined with the API 20NE microtest system, the following characteristics are present: nitrate reduction, esculin hydrolysis, assimilation of glucose, arabinose (weakly), mannose, mannitol, N-acetyl-glucosamine, potassium gluconate, adipic acid, malate, trisodium citrate and phenylacetic acid, but not indol production from L-tryptophane, fermentation of glucose, activity of arginine dihydrolase, urease and beta-galactosidase (PNPG), gelatin liquefaction and assimilation of maltose and capric acid.

When examined with the API ZYM microtest system, the following characteristics are present: alkaline phosphatase (weakly), butyrate esterase (C4) (weakly), caprylate esterase lipase (C8) (weakly), leucyl arylamidase, acid phosphatase (weakly) and phosphoamidase (weakly) but not myristate lipase (C14), valine arylamidase, cystin arylamidase, trypsin, chymotrypsin, alpha-galactosidase, beta-galactosidase, beta-glucuronidase, alpha-glucosidase, beta-glucosidase, N-acetyl-beta-glucosaminidase, alpha-mannosidase and alpha-fucosidase.

The type strain, LMG 31836^T^ (=CECT 30335), was isolated in 2018 from forest soil. The whole-genome sequence of strain LMG 31836^T^ has a size of 9.27 Mbp. The G+C content is 61.59 mol%. The 16S rRNA gene, *recA* gene and whole-genome sequences of strain LMG 31836^T^ are publicly available under accession numbers HG995124, HG995055 and CAJNBO000000000, respectively.

### 5.3. Description of Paraburkholderia haematera *sp.* nov.

*Paraburkholderia haematera* sp. nov. (hae.ma.te’ra. Gr. adj. *haemateros*, blood-thirsty; N.L. fem. adj. *haematera*, blood-thirsty; because of its hemolytic behavior on horse blood agar).

Cells are non-motile, rod-shaped, approximately 1 µm long and 0.5 µm wide. After incubation on NAB at 28 °C for three days, colonies have a diameter of 1 mm and are circular, opaque and beige. They are flat or slightly raised and have an entire edge and a smooth surface. Strain LMG 31837^T^ optimally grows on NAB and TSA between 15 and 28 °C in aerobic conditions, but does not grow in anaerobic conditions on NAB, TSA or TSA supplemented with 10 mM KNO_3_. No growth at 4, 37, 40 and 45 °C. Growth in the presence of 0% and 1% NaCl, but not 2% or more. Growth at pH 6.0 and 7.0, but not at 4.0, 5.0, 8.0 or 9.0. Growth on MacConkey and Drigalski agar and no growth on cetrimide agar. Growth on Tween 20, 40, 60 and 80 agar base and hydrolysis of all Tweens after 7 days of incubation. Hemolysis on horse blood agar but no DNase activity and hydrolysis of starch or casein. Oxidase and weak catalase activity is present.

When examined with the API 20NE microtest system, the following characteristics are present: esculin hydrolysis, assimilation of glucose, arabinose (weakly), mannose, mannitol, N-acetyl-glucosamine, potassium gluconate, adipic acid (weakly), malate, trisodium citrate and phenylacetic acid but not nitrate reduction, indol production from L-tryptophane, fermentation of glucose, activity of arginine dihydrolase, urease and beta-galactosidase (PNPG), gelatin liquefaction and assimilation of maltose and capric acid.

When examined with the API ZYM microtest system, the following characteristics are present: alkaline phosphatase (weakly), butyrate esterase (C4) (weakly), caprylate esterase lipase (C8) (weakly), leucyl arylamidase, acid phosphatase and phosphoamidase (weakly) but not myristate lipase (C14), valine arylamidase, cystin arylamidase, trypsin, chymotrypsin, alpha-galactosidase, beta-galactosidase, beta-glucuronidase, alpha-glucosidase, beta-glucosidase, N-acetyl-beta-glucosaminidase, alpha-mannosidase and alpha-fucosidase.

The type strain is LMG 31837^T^ (=CECT 30336) and was isolated in 2016 from forest soil. The whole-genome sequence of strain LMG 31837^T^ has a size of 7.82 Mbp. The G+C content is 61.71 mol%. The 16S rRNA gene, *recA* gene and whole-genome sequences of strain LMG 31837^T^ are publicly available under accession numbers HG995125, HG995035 and CAJNBK000000000, respectively.

### 5.4. Emended Description of Paraburkholderia sediminicola Lim et al. 2008

The description is as before [83] with the following addition. The G+C content of the type strain genome is 61.4 mol%, its approximate size is 8.64 Mbp. The whole-genome sequence of strain LMG 24238^T^ is available under accession number CADIKC000000000.

## Figures and Tables

**Table 1:** Overview of the isolates studied. Isolates marked with an asterisk were selected for whole genome sequence analysis.

Strain	Source	Country	Year	Isolation Medium	Reference
*Paraburkholderia aspalathi*					
R-20943 (=Hg8) *	Coal tar-contaminated hillside soil	United States	Unknown	/	Wilson et al. [37]
R-69658 *	Forest topsoil	Belgium	2016	DNG	This study
R-69746 *	Forest topsoil	Belgium	2016	DNG	This study
R-69781	Forest topsoil	Belgium	2016	DNG	This study
R-69848	Forest topsoil	Belgium	2016	DNG	This study
R-69955	Forest topsoil	Belgium	2016	DNG	This study
R-70036	Forest topsoil	Belgium	2016	DNG	This study
R-75464	Forest topsoil	Belgium	2018	BCEM	This study
R-75465 *	Forest topsoil	Belgium	2018	BCEM	This study
*Paraburkholderia nemoris* sp. nov.					
LMG 31836^T^ *	Forest topsoil	Belgium	2018	BCEM	This study
LMG 22931 (=G47-12) *	Grassland soil	The Netherlands	2003	/	Salles et al. [40]
LMG 31840 *	Forest topsoil	Belgium	2016	DNG	This study
R-69608 *	Forest topsoil	Belgium	2016	DNG	This study
R-69616	Forest topsoil	Belgium	2016	DNG	This study
R-69643	Forest topsoil	Belgium	2016	DNG	This study
R-69776 *	Forest topsoil	Belgium	2016	DNG	This study
R-69861	Forest topsoil	Belgium	2016	DNG	This study
R-70025	Forest topsoil	Belgium	2016	DNG	This study
R-74083	Forest topsoil	Belgium	2018	Pikovskaya	This study
R-74093	Forest topsoil	Belgium	2018	Pikovskaya	This study
R-75460	Forest topsoil	Belgium	2018	BCEM	This study
R-75463	Forest topsoil	Belgium	2018	BCEM	This study
R-75466	Forest topsoil	Belgium	2018	BCEM	This study
R-75776	Forest topsoil	Belgium	2017	PCAT	This study
R-75777 *	Forest topsoil	Belgium	2017	PCAT	This study
R-75793	Forest topsoil	Belgium	2017	PCAT	This study
R-75795	Forest topsoil	Belgium	2017	PCAT	This study
R-75796	Forest topsoil	Belgium	2017	PCAT	This study
R-75797	Forest topsoil	Belgium	2017	PCAT	This study
R-75798	Forest topsoil	Belgium	2017	PCAT	This study
R-75799	Forest topsoil	Belgium	2017	PCAT	This study
R-75800	Forest topsoil	Belgium	2017	PCAT	This study
R-75801	Forest topsoil	Belgium	2017	PCAT	This study
R-75803	Forest topsoil	Belgium	2017	PCAT	This study
*Paraburkholderia domus* sp. nov.					
LMG 31832^T^ *	Forest topsoil	Belgium	2018	BCEM	This study
LMG 19510 (=DSM 6431)	Industrial waste deposit soil	Germany	Unknown	/	Schindowski et al. [38]
R-23782	Succulent soil (porch)	Belgium	2003	/	Vanlaere et al. [39]
R-69749 *	Forest topsoil	Belgium	2016	DNG	This study
R-69927 *	Forest topsoil	Belgium	2016	DNG	This study
R-69973	Forest topsoil	Belgium	2016	DNG	This study
R-69977	Forest topsoil	Belgium	2016	DNG	This study
R-69980	Forest topsoil	Belgium	2016	DNG	This study
R-69981	Forest topsoil	Belgium	2016	DNG	This study
R-70006 *	Forest topsoil	Belgium	2016	DNG	This study
R-70199 *	Forest topsoil	Belgium	2016	DNG	This study
R-70211 *	Forest topsoil	Belgium	2016	DNG	This study
R-75306	Forest topsoil	Belgium	2018	Pikovskaya	This study
R-75471 *	Forest topsoil	Belgium	2018	BCEM	This study
R-75473	Forest topsoil	Belgium	2018	BCEM	This study
R-75474	Forest topsoil	Belgium	2018	BCEM	This study
R-75475	Forest topsoil	Belgium	2018	BCEM	This study
R-75476	Forest topsoil	Belgium	2018	BCEM	This study
R-75477	Forest topsoil	Belgium	2018	BCEM	This study
R-75478	Forest topsoil	Belgium	2018	BCEM	This study
R-75479	Forest topsoil	Belgium	2018	BCEM	This study
R-75480	Forest topsoil	Belgium	2018	BCEM	This study
R-75481	Forest topsoil	Belgium	2018	BCEM	This study
R-75482	Forest topsoil	Belgium	2018	BCEM	This study
R-75484	Forest topsoil	Belgium	2018	BCEM	This study
R-75485	Forest topsoil	Belgium	2018	BCEM	This study
R-75486	Forest topsoil	Belgium	2018	BCEM	This study
R-75487	Forest topsoil	Belgium	2018	BCEM	This study
R-75488	Forest topsoil	Belgium	2018	BCEM	This study
R-75589	Forest topsoil	Belgium	2018	BCEM	This study
R-75773	Forest topsoil	Belgium	2017	PCAT	This study
R-75774	Forest topsoil	Belgium	2017	PCAT	This study
R-75784	Forest topsoil	Belgium	2017	PCAT	This study
R-75788	Forest topsoil	Belgium	2017	PCAT	This study
R-75789	Forest topsoil	Belgium	2017	PCAT	This study
R-75804	Forest topsoil	Belgium	2017	PCAT	This study
*Paraburkholderia haematera* sp. nov.					
LMG 31837^T^ *	Forest topsoil	Belgium	2016	DNG	This study

BCEM, *B. cepacia* complex enrichment medium; DNG, 1/100 diluted nutrient broth solidified with Gelzan CM; PCAT, 1/10 diluted *Pseudomonas cepacia* azaleic acid tryptamine medium; Pikovskaya, modified Pikovskaya medium.

**Table 2:** Pairwise dDDH (%) values between strains of the present study and their closest phylogenetic relatives. Values of 70% or higher were highlighted in color according to their respective clusters (Figure 2); A, blue; B, green; C, red.

	R-20943	R-69658	R-69746	R-75465	LMG 31836^T^	LMG 22931	LMG 31840	R-69608	R-69776	R-75777	LMG 31832^T^	R-69749	R-69927	R-70006	R-70199	R-70211	R-75471	LMG 31837^T^
*P. aspalathi* LMG 27731^T^	84.5	82.9	85.0	85.2	62.6	63.0	63.0	63.0	62.8	62.8	40.6	40.6	40.6	40.5	40.6	40.7	40.5	39.3
*P. madseniana* RP11^T^	57.4	57.3	57.1	56.7	57.3	57.6	57.5	57.5	57.0	57.4	41.9	42.0	41.9	41.9	41.9	41.9	41.9	41.0
*P. sediminicola* LMG 24238^T^	55.7	55.8	55.7	55.7	55.5	55.6	55.5	55.4	55.4	55.5	39.9	40.1	40.0	40.0	39.9	40.0	40.0	38.9
*P. solitsugae* 1N^T^	41.4	41.4	41.3	41.3	41.2	41.4	41.4	41.4	41.2	41.4	46.3	46.3	46.4	46.4	46.4	46.4	46.5	41.0
*P. elongata* 5N^T^	42.3	42.6	42.6	42.6	42.7	43.0	42.9	42.9	42.6	42.8	43.8	43.8	43.7	43.7	43.8	43.8	43.8	44.8
*P. caffeinilytica* CF1^T^	43.2	43.2	43.3	43.4	44.0	44.0	43.8	43.9	43.7	43.9	39.8	39.8	39.7	39.7	39.8	39.8	39.8	39.1
R-20943	/	83.4	84.9	83.9	63.2	63.4	63.2	63.3	63.3	63.1	40.4	40.6	40.4	40.4	40.4	40.5	40.4	39.3
R-69658	83.4	/	83.0	82.9	63.9	64.9	65.3	64.6	65.1	65.0	40.7	40.8	40.7	40.7	40.9	40.7	40.8	39.4
R-69746	84.9	83.0	/	85.3	62.7	63.0	63.2	63.3	63.0	63.1	40.8	41.0	40.9	40.8	40.8	40.6	40.8	39.6
R-75465	83.9	82.9	85.3	/	62.9	63.0	63.2	62.8	62.8	62.9	40.6	40.9	40.7	40.7	40.7	40.6	40.6	39.3
LMG 31836^T^	63.2	63.9	62.7	62.9	/	90.4	89.2	90.6	89.5	90.1	40.7	41.0	40.6	40.6	40.7	40.7	40.6	39.5
LMG 22931	63.4	64.9	63.0	63.0	90.4	/	89.4	89.6	89.7	90.1	40.9	41.3	40.8	40.9	40.9	40.8	40.9	39.7
LMG 31840	63.2	65.3	63.2	63.2	89.2	89.4	/	91.4	91.2	91.6	41.2	43.7	41.1	41.1	41.2	40.8	41.1	39.7
R-69608	63.3	64.6	63.3	62.8	90.6	89.6	91.4	/	91.9	91.6	41.2	43.5	41.1	41.1	41.2	40.9	41.1	39.8
R-69776	63.3	65.1	63.0	62.8	89.5	89.7	91.2	91.9	/	98.0	41.1	43.9	41.1	41.1	41.1	40.7	41.1	39.7
R-75777	63.1	65.0	63.1	62.9	90.1	90.1	91.6	91.6	98.0	/	40.9	43.5	40.9	40.9	40.9	40.8	40.9	39.8
LMG 31832^T^	40.4	40.7	40.8	40.6	40.7	40.9	41.2	41.2	41.1	40.9	/	95.1	95.2	96.1	96.2	96.1	96.6	40.7
R-69749	40.6	40.8	41.0	40.9	41.0	41.3	43.7	43.5	43.9	43.5	95.1	/	96.6	97.0	97.6	97.5	97.5	40.8
R-69927	40.4	40.7	40.9	40.7	40.6	40.8	41.1	41.1	41.1	40.9	95.2	96.6	/	97.1	97.8	97.8	98.1	40.6
R-70006	40.4	40.7	40.8	40.7	40.6	40.9	41.1	41.1	41.1	40.9	96.1	97.0	97.1	/	98.1	97.7	98.4	40.6
R-70199	40.4	40.9	40.8	40.7	40.7	40.9	41.2	41.2	41.1	40.9	96.2	97.6	97.8	98.1	/	98.4	98.5	40.5
R-70211	40.5	40.7	40.6	40.6	40.7	40.8	40.8	40.9	40.7	40.8	96.1	97.5	97.8	97.7	98.4	/	98.5	40.4
R-75471	40.4	40.8	40.8	40.6	40.6	40.9	41.1	41.1	41.1	40.9	96.6	97.5	98.1	98.4	98.5	98.5	/	40.5
LMG 31837^T^	39.3	39.4	39.6	39.3	39.5	39.7	39.7	39.8	39.7	39.8	40.7	40.8	40.6	40.6	40.5	40.4	40.5	/

**Table 3:** Pairwise orthoANIu (%) values between strains of the present study and their closest phylogenetic relatives. Values of 96% or higher were highlighted in color according to their respective clusters (Figure 2); A, blue; B, green; C, red.

	R-20943	R-69658	R-69746	R-75465	LMG 31836^T^	LMG 22931	LMG 31840	R-69608	R-69776	R-75777	LMG 31832^T^	R-69749	R-69927	R-70006	R-70199	R-70211	R-75471	LMG 31837^T^
*P. aspalathi* LMG 27731^T^	98.2	98.0	98.3	98.3	95.1	95.2	95.2	95.3	95.2	95.2	89.9	89.8	90.0	89.8	89.8	89.9	89.9	89.7
*P. madseniana* RP11^T^	94.2	94.2	94.2	94.1	94.1	94.3	94.2	94.3	94.2	94.3	90.2	90.3	90.2	90.2	90.2	90.2	90.3	90.1
*P. sediminicola* LMG 24238^T^	94.0	94.0	94.0	93.9	93.8	93.9	94.0	93.9	93.9	93.9	89.6	89.7	89.7	89.7	89.6	89.6	89.6	89.3
*P. solitsugae* 1N^T^	90.1	90.0	90.0	90.0	90.1	90.2	90.1	90.3	90.1	90.3	91.5	91.5	91.5	91.6	91.5	91.6	91.6	90.0
*P. elongata* 5N^T^	90.4	90.5	90.5	90.5	90.7	90.8	90.6	90.6	90.6	90.6	90.8	90.7	90.8	90.7	90.7	90.8	90.8	91.2
*P. caffeinilytica* CF1^T^	90.7	90.8	90.7	90.8	90.9	91.0	90.9	90.9	90.9	91.0	89.6	89.6	89.6	89.5	89.6	89.5	89.6	89.4
R-20943	/	98.0	98.2	98.1	95.2	95.4	95.3	95.3	95.3	95.3	89.8	89.8	89.9	89.7	89.8	89.9	89.8	89.5
R-69658	98.0	/	98.0	98.1	95.4	95.6	95.6	95.5	95.6	95.6	89.9	89.8	89.9	89.9	89.9	89.8	89.9	89.5
R-69746	98.2	98.0	/	98.3	95.1	95.2	95.3	95.3	95.3	95.3	89.8	89.8	89.8	89.8	89.8	89.8	89.9	89.7
R-75465	98.1	98.1	98.3	/	95.2	95.3	95.3	95.2	95.2	95.3	89.9	90.0	89.9	89.8	89.9	89.9	89.9	89.5
LMG 31836^T^	95.2	95.4	95.1	95.2	/	98.9	98.7	98.9	98.7	98.8	89.9	89.9	89.7	89.9	89.7	89.9	89.8	89.6
LMG 22931	95.4	95.6	95.2	95.3	98.9	/	98.7	98.8	98.7	98.8	89.9	90.1	90.0	90.0	90.1	89.9	89.9	89.7
LMG 31840	95.3	95.6	95.3	95.3	98.7	98.7	/	99.0	98.9	99.0	90.1	90.9	90.0	90.0	90.1	89.9	90.1	89.7
R-69608	95.3	95.5	95.3	95.2	98.9	98.8	99.0	/	99.0	99.0	90.0	90.8	90.0	90.0	90.0	89.9	90.1	89.8
R-69776	95.3	95.6	95.3	95.2	98.7	98.7	98.9	99.0	/	99.7	90.0	90.9	90.0	89.9	90.0	90.0	90.1	89.6
R-75777	95.3	95.6	95.3	95.3	98.8	98.8	99.0	99.0	99.7	/	89.9	90.8	89.9	89.9	89.9	89.9	90.0	89.8
LMG 31832^T^	89.8	89.9	89.8	89.9	89.9	89.9	90.1	90.0	90.0	89.9	/	99.4	99.4	99.5	99.5	99.5	99.5	90.0
R-69749	89.8	89.8	89.8	90.0	89.9	90.1	90.9	90.8	90.9	90.8	99.4	/	99.6	99.6	99.6	99.6	99.7	90.1
R-69927	89.9	89.9	89.8	89.9	89.7	90.0	90.0	90.0	90.0	89.9	99.4	99.6	/	99.5	99.6	99.6	99.7	90.1
R-70006	89.7	89.9	89.8	89.8	89.9	90.0	90.0	90.0	89.9	89.9	99.5	99.6	99.5	/	99.7	99.7	99.8	90.1
R-70199	89.8	89.9	89.8	89.9	89.7	90.1	90.1	90.0	90.0	89.9	99.5	99.6	99.6	99.7	/	99.7	99.8	90.1
R-70211	89.9	89.8	89.8	89.9	89.9	89.9	89.9	89.9	90.0	89.9	99.5	99.6	99.6	99.7	99.7	/	99.8	90.0
R-75471	89.8	89.9	89.9	89.9	89.8	89.9	90.1	90.1	90.1	90.0	99.5	99.7	99.7	99.8	99.8	99.8	/	90.1
LMG 31837^T^	89.5	89.5	89.7	89.5	89.6	89.7	89.7	89.8	89.6	89.8	90.0	90.1	90.1	90.1	90.1	90.0	90.1	/

**Table 4:** Genome features and accession numbers of the final genome assemblies of *P. sediminicola* LMG 24238^T^ and of the 18 isolates of the present study.

	Genome Size (Mb)	Mol % G+C	No. of Contigs	N50 (kb)	No. of CDS	No. of tRNA Genes	No. of rRNA Genes	Accession Number
LMG 24238^T^	8.64	61.40	40	513.2	7690	60	2	CADIKC000000000
R-20943	8.74	61.34	39	857.6	7831	60	3	CAJNBA000000000
R-69658	9.05	61.43	380	70.9	8212	60	2	CAJNAU000000000
R-69746	9.66	61.25	520	61.7	8837	61	3	CAJNBE000000000
R-75465	9.50	61.28	387	92.4	8623	60	3	CAJNAX000000000
LMG 31836^T^	9.27	61.59	128	261.5	8438	64	2	CAJNBO000000000
LMG 22931	8.64	61.47	68	263.0	7692	62	3	CAJNBC000000000
LMG 31840	8.85	61.40	152	170.6	7822	60	4	CAJNBI000000000
R-69608	8.91	61.40	116	252.5	7898	61	3	CAJNAW000000000
R-69776	9.28	61.30	113	288.6	8167	62	2	CAJNBH000000000
R-75777	9.08	61.33	98	320.9	8045	61	3	CAJNBN000000000
LMG 31832^T^	8.75	61.32	99	229.8	7830	57	3	CAJNAT000000000
R-69749	9.46	61.14	147	210.7	8462	57	2	CAJNAY000000000
R-69927	8.71	61.35	133	205.4	7784	59	3	CAJNAV000000000
R-70006	9.16	61.19	115	208.5	8250	59	2	CAJNBD000000000
R-70199	9.06	61.24	124	265.3	8195	62	3	CAJNBB000000000
R-70211	8.63	61.28	91	323.0	7680	59	2	CAJNAS000000000
R-75471	8.19	61.42	83	359.9	7292	56	2	CAJNAZ000000000
LMG 31837^T^	7.82	61.71	76	270.9	6967	58	2	CAJNBK000000000

**Table 5:** Overview of enzymes required for the degradation of benzoic acid, 3-hydroxybenzoic acid, lignin-derived compounds ferulic acid and p-coumaric acid and their degradation intermediates in the draft genomes of the 18 selected isolates and their closest relatives *P. aspalathi* LMG 27731^T^, *P. madseniana* RP11^T^, *P. sediminicola* LMG 24238^T^, *P. solitsugae* 1N^T^, *P. elongata* 5N^T^ and *P. caffeinilytica* CF1^T^. +, coding sequence present; −, coding sequence absent.

	LMG 31836^T^ (cluster B)	LMG 22931 (cluster B)	LMG 31840 (cluster B)	R-69608 (cluster B)	R-69776 (cluster B)	R-75777 (cluster B)	LMG 31832^T^ (cluster C)	R-69749 (cluster C)	R-69927 (cluster C)	R-70006 (cluster C)	R-70199 (cluster C)	R-70211 (cluster C)	R-75471 (cluster C)	LMG 31837^T^	*P. aspalathi* LMG 27731^T^	*P. aspalathi* R-20943	*P. aspalathi* R-69658	*P. aspalathi* R-69746	*P. aspalathi* R-75465	*P. madseniana* RP11^T^	*P. sediminicola* LMG 24238^T^	*P. solitsugae* 1N^T^	*P. elongata* 5N^T^	*P. caffeinilytica* CF1^T^
**Catechol (meta-cleavage pathway)**																								
Catechol 2,3 dioxygenase (EC 1.13.11.2)	−	−	+	+	+	+	+	+	+	+	+	−	+	−	−	+	−	−	−	−	−	−	+	−
2-Hydroxymuconic semialdehyde hydrolase (EC 3.7.1.9)	−	−	−	−	−	−	−	−	+	−	−	−	−	−	−	+	−	−	−	−	−	−	−	−
2-Oxopent-4-enoate hydratase (EC 4.2.1.80)	−	−	−	+	−	−	+	+	+	+	+	+	+	−	−	−	−	−	−	+	+	+	+	−
4-Hydroxy-2-oxovalerate aldolase (EC 4.1.3.39)	+	−	−	+	+	+	+	+	+	+	+	+	+	+	+	+	+	−	+	−	+	+	+	+
**Catechol (ortho-cleavage pathway)**																								
Catechol 1,2 dioxygenase (EC 1.13.11.1)	+	+	+	+	+	+	+	+	+	+	+	+	+	+	+	+	+	+	+	+	+	+	+	+
Muconate cycloisomerase (EC 5.5.1.1)	−	−	+	+	+	+	+	+	+	+	+	+	+	+	+	−	+	+	+	+	+	+	+	+
Muconolactone isomerase (EC 5.3.3.4)	+	+	+	+	+	+	+	+	+	+	+	+	+	+	+	+	+	+	+	+	+	+	+	+
3-Oxoadipate enol-lactonase (EC 3.1.1.24)	+	+	+	+	+	+	+	+	+	+	+	+	+	+	+	+	+	+	+	+	+	+	+	+
**Ferulic acid to vanillin**																								
Feruloyl-CoA synthase (EC 6.2.1.34)	+	+	+	+	+	+	+	+	+	+	+	+	+	+	+	+	+	+	+	+	+	+	+	−
Feruloyl-CoA hydratase/lyase (EC 4.1.2.61)	+	+	+	+	+	+	+	+	+	+	+	+	+	+	+	+	+	+	+	+	+	+	+	−
**Vanillin to protocatechuate**																								
Vanillin dehydrogenase (EC 1.2.1.67)	+	+	+	+	+	+	+	+	+	+	+	+	+	+	+	+	+	+	+	−	+	+	−	−
Vanillate monooxygenase (EC 1.14.13.82)	−	−	−	−	−	−	−	−	−	−	−	−	−	−	−	−	−	−	−	−	−	−	−	+
Vanillate/3-O-methylgallate O-demethylase (EC 2.1.1.341)	−	−	−	−	−	−	−	−	−	−	−	−	−	−	−	−	−	−	−	−	−	−	−	−
**p-Coumaric acid to protocatechuate**																								
4-Coumarate-CoA ligase (EC 6.2.1.12)	−	−	−	−	−	−	−	−	−	−	−	−	−	−	−	−	−	−	−	−	−	−	−	−
Feruloyl-CoA hydratase/lyase (EC 4.1.2.61)	+	+	+	+	+	+	+	+	+	+	+	+	+	+	+	+	+	+	+	+	+	+	+	−
Benzaldehyde dehydrogenase (EC 1.2.1.28)	+	+	+	+	+	+	−	−	−	−	−	−	−	+	+	+	+	+	+	+	+	+	+	+
4-Hydroxybenzoate 3-monooxygenase (EC 1.14.13.2)	+	+	+	+	+	+	+	+	+	+	+	+	+	+	+	+	+	+	+	+	+	+	+	+
**Protocatechuate (meta-cleavage pathway)**																								
Protocatechuate 4,5-dioxygenase (EC 1.13.11.8)	+	+	+	+	+	+	+	+	+	+	+	+	+	+	+	+	+	+	+	+	+	+	+	−
2-Hydroxy-4-carboxymuconate 6-semialdehyde dehydrogenase (EC 1.1.1.312)	−	+	−	−	−	−	+	+	+	+	+	+	+	−	−	−	−	−	−	−	−	+	−	−
2-Pyrone-4,6-dicarboxylate hydrolase (EC 3.1.1.57)	−	+	−	−	+	+	+	+	+	+	+	+	+	+	−	−	−	−	−	−	−	+	−	−
4-Oxalomesaconate hydratase (EC 4.2.1.83)	+	+	+	+	+	+	+	+	+	+	+	+	+	+	+	+	+	+	+	+	+	+	+	+
4-Carboxy-4-hydroxy-2-oxoadipate aldolase (EC 4.1.3.17)	+	+	+	+	+	+	+	+	+	+	+	+	+	+	+	+	+	+	+	+	+	+	+	−
**3-Hydroxybenzoic acid**																								
3-Hydroxybenzoate 6-monooxygenase (EC 1.14.13.24)	−	−	−	−	−	−	−	−	−	−	−	−	−	−	−	−	−	−	−	−	−	−	+	−
Gentisate 1,2-dioxygenase (EC 1.13.11.4)	−	−	−	−	−	−	−	−	−	−	−	−	−	−	−	+	−	+	+	+	−	+	+	−
Maleylacetoacetate isomerase (EC 5.2.1.2)	+	+	+	+	+	+	+	+	+	+	+	+	+	+	+	+	+	+	+	+	+	+	+	+
3-Fumarylpyruvate hydrolase (EC 3.7.1.20)	+	+	+	+	+	+	+	+	+	+	+	+	+	+	+	+	+	+	+	+	+	+	+	+

**Table 6:** Differential phenotypic characteristics of strains LMG 31836^T^, LMG31832^T^, LMG 31837^T^ and closely related type strains *P. aspalathi* LMG 27731^T^, *P. madseniana* RP11^T^, *P. sediminicola* LMG 24238^T^, *P. solitsugae* 1N^T^, *P. elongata* 5N^T^ and *P. caffeinilytica* CF1^T^. All data are from the present study. +, positive reaction; w, weakly positive reaction; −, negative reaction; (d), delayed reaction. Results after 3 and 7 days are represented (3 days/7 days). Results of API 20NE and API ZYM microtests were read after 2 days and 4 h of incubation, respectively.

Characteristic	LMG 31836^T^	LMG 31832^T^	LMG 31837^T^	*P. aspalathi* LMG 27731^T^	*P. madseniana* RP11^T^	*P. sediminicola* LMG 24238^T^	*P. solitsugae* 1N^T^	*P. elongata* 5N^T^	*P. caffeinilytica* CF1^T^
Hemolysis on horse blood agar	w/+	w/+	w/+	−/+	−/+	−/+	−/w	−/−	−/+
Growth in the presence of 1% NaCl	+/+	+/+	+/+	+/+	+/+	−/−	−/−	−/−	−/+
Oxidase activity	+	+	+	+	+	+	w (d)	w (d)	+ (d)
Catalase activity	w (d)	w	w	+	w	+	+	w (d)	w
Lipase activity on:									
Tween 20	w/+	w/+	w/+	+/+	+/+	−/+	+/+	+/+	−/+
Tween 40	+/+	w/+	w/+	−/+	−/+	−/+	+/+	−/−	−/+
Tween 60	+/+	−/+	−/+	−/+	+/+	+/+	+/+	w/w	+/+
Tween 80	w/+	+/+	+/+	+/+	+/+	−/+	+/+	w/w	+/+
API 20NE:									
Nitrate reduction	+	+	−	−	−	−	−	−	−
Beta-galactosidase (PNPG)	−	−	−	w	−	w	−	−	−
Assimilation of (API 20NE):									
Arabinose	w	w	w	+	w	+	+	+	+
N-acetyl-glucosamine	+	−	+	+	+	+	+	+	+
Maltose	−	−	−	−	−	−	−	−	w
Adipic acid	+	w	w	+	+	+	+	+	w
Trisodium citrate	+	−	+	+	+	+	−	w	+
Phenylacetic acid	+	−	+	+	+	+	+	+	+
API ZYM:									
Alkaline phosphatase	w	w	w	+	w	+	w	w	+
Phosphoamidase	w	+	+	+	w	+	+	+	+

## Data Availability

Gene and genome sequences were deposited in the European Nucleotide Archive (ENA). The *recA* and 16S rRNA gene sequences, raw reads and annotated assemblies can be found via the BioProject accession numbers PRJEB41851 and PRJEB37806, and the accession numbers listed in Figure 1, Figure 2 and Table 4.

## Supplementary Materials

The following are available online at https://www.mdpi.com/article/10.3390/ijms22137003/s1.
