# Supplementary material for: Genomic Aromatic Compound Degradation Potential of Novel Paraburkholderia Species: Paraburkholderia domus sp. nov., Paraburkholderia haematera sp. nov. and Paraburkholderia nemoris sp. nov
Source: Int J Mol Sci. 2021 Jun 29;22(13):7003. doi: 10.3390/ijms22137003 (PMC8268980; doi:10.3390/ijms22137003)
Supplement: Supplementary file 1 [file ijms-22-07003-s001.zip › ijms-1237573-supplementary.pdf]

Supplementary table S1. Pairwise dDDH values (formula d_4) and their respective confidence intervals between the draft genomes of the 18 selected isolates and their closest neighbors.

Strain 1	Strain 2	dDDH (%)	C.I. (%)	Strain 1	Strain 2	dDDH (%)	C.I. (%)
R-20943	*P. aspalathi* LMG 27731T	84.5	[81.7 - 86.9]	R-69746	R-75465	85.3	[82.5 - 87.6]
R-20943	*P. madseniana* RP11T	57.4	[54.6 - 60.1]	R-69746	LMG 31836T	62.7	[59.9 - 65.6]
R-20943	*P. sediminicola* LMG 24238T	55.7	[52.9 - 58.4]	R-69746	R-69776	63	[60.1 - 65.9]
R-20943	*P. solitsugae* 1NT	41.4	[38.9 - 44.0]	R-69746	R-75777	63.1	[60.2 - 65.9]
R-20943	*P. elongata* 5NT	42.3	[39.8 - 44.9]	R-69746	LMG 31832T	40.8	[38.3 - 43.4]
R-20943	*P. caffeinilytica* CF1T	43.2	[40.7 - 45.8]	R-69746	R-69749	41	[38.5 - 43.5]
R-20943	R-69658	83.4	[80.6 - 85.9]	R-69746	R-69927	40.9	[38.4 - 43.4]
R-20943	R-69746	84.9	[82.2 - 87.3]	R-69746	R-70006	40.8	[38.3 - 43.4]
R-20943	R-75465	83.9	[81.1 - 86.4]	R-69746	R-70199	40.8	[38.3 - 43.3]
R-20943	LMG 31836T	63.2	[60.3 - 66.0]	R-69746	R-70211	40.6	[38.2 - 43.2]
R-20943	LMG 31840	63.2	[60.3 - 66.0]	R-69746	R-75471	40.8	[38.3 - 43.4]
R-20943	R-69608	63.3	[60.4 - 66.1]	R-69746	LMG 31837T	39.6	[37.1 - 42.1]
R-20943	R-69776	63.3	[60.4 - 66.1]	R-75465	*P. aspalathi* LMG 27731T	85.2	[82.5 - 87.6]
R-20943	R-75777	63.1	[60.2 - 65.9]	R-75465	*P. madseniana* RP11T	56.7	[53.9 - 59.4]
R-20943	LMG 31832T	40.4	[37.9 - 42.9]	R-75465	*P. sediminicola* LMG 24238T	55.7	[53.0 - 58.4]
R-20943	R-69749	40.6	[38.1 - 43.1]	R-75465	*P. solitsugae* 1NT	41.3	[38.8 - 43.9]
R-20943	R-69927	40.4	[37.9 - 43.0]	R-75465	*P. elongata* 5NT	42.6	[40.1 - 45.1]
R-20943	R-70006	40.4	[37.9 - 42.9]	R-75465	*P. caffeinilytica* CF1T	43.4	[40.9 - 45.9]
R-20943	R-70199	40.4	[38.0 - 43.0]	R-75465	R-75777	62.9	[60.0 - 65.7]
R-20943	R-70211	40.5	[38.0 - 43.0]	R-75465	LMG 31832T	40.6	[38.1 - 43.2]
R-20943	R-75471	40.4	[37.9 - 43.0]	R-75465	R-75471	40.6	[38.1 - 43.2]
R-20943	LMG 31837T	39.3	[36.8 - 41.8]	LMG 31836T	*P. aspalathi* LMG 27731T	62.6	[59.7 - 65.4]
R-69658	*P. aspalathi* LMG 27731T	82.9	[80.1 - 85.4]	LMG 31836T	*P. madseniana* RP11T	57.3	[54.6 - 60.1]
R-69658	*P. madseniana* RP11T	57.3	[54.5 - 60.0]	LMG 31836T	*P. sediminicola* LMG 24238T	55.5	[52.7 - 58.2]
R-69658	*P. sediminicola* LMG 24238T	55.8	[53.0 - 58.5]	LMG 31836T	*P. solitsugae* 1NT	41.2	[38.7 - 43.8]
R-69658	*P. solitsugae* 1NT	41.4	[38.9 - 44.0]	LMG 31836T	*P. elongata* 5NT	42.7	[40.2 - 45.3]
R-69658	*P. elongata* 5NT	42.6	[40.1 - 45.1]	LMG 31836T	*P. caffeinilytica* CF1T	44	[41.4 - 46.5]
R-69658	*P. caffeinilytica* CF1T	43.2	[40.7 - 45.8]	LMG 31836T	R-75465	62.9	[60.0 - 65.7]
R-69658	R-69746	83	[80.1 - 85.5]	LMG 31836T	R-75777	90.1	[87.8 - 92.0]
R-69658	R-75465	82.9	[80.1 - 85.4]	LMG 31836T	LMG 31832T	40.7	[38.2 - 43.2]
R-69658	LMG 31836T	63.9	[60.9 - 66.7]	LMG 31836T	R-75471	40.6	[38.2 - 43.2]
R-69658	R-69776	65.1	[62.2 - 67.9]	LMG 22931	*P. aspalathi* LMG 27731T	63	[60.1 - 65.9]
R-69658	R-75777	65	[62.1 - 67.8]	LMG 22931	*P. madseniana* RP11T	57.6	[54.8 - 60.4]
R-69658	LMG 31832T	40.7	[38.2 - 43.2]	LMG 22931	*P. sediminicola* LMG 24238T	55.6	[52.9 - 58.3]
R-69658	R-69749	40.8	[38.3 - 43.3]	LMG 22931	*P. solitsugae* 1NT	41.4	[38.9 - 43.9]
R-69658	R-69927	40.7	[38.2 - 43.2]	LMG 22931	*P. elongata* 5NT	43	[40.5 - 45.6]
R-69658	R-70006	40.7	[38.2 - 43.2]	LMG 22931	*P. caffeinilytica* CF1T	44	[41.5 - 46.6]
R-69658	R-70199	40.9	[38.4 - 43.4]	LMG 22931	R-20943	63.4	[60.5 - 66.2]
R-69658	R-70211	40.7	[38.2 - 43.3]	LMG 22931	R-69658	64.9	[62.0 - 67.8]
R-69658	R-75471	40.8	[38.3 - 43.3]	LMG 22931	R-69746	63	[60.1 - 65.8]
R-69658	LMG 31837T	39.4	[37.0 - 42.0]	LMG 22931	R-75465	63	[60.1 - 65.8]
R-69746	*P. aspalathi* LMG 27731T	85	[82.3 - 87.4]	LMG 22931	LMG 31836T	90.4	[88.1 - 92.3]
R-69746	*P. madseniana* RP11T	57.1	[54.3 - 59.8]	LMG 22931	LMG 31840	89.4	[87.1 - 91.4]
R-69746	*P. sediminicola* LMG 24238T	55.7	[53.0 - 58.4]	LMG 22931	R-69608	89.6	[87.2 - 91.6]
R-69746	*P. solitsugae* 1NT	41.3	[38.8 - 43.8]	LMG 22931	R-69776	89.7	[87.3 - 91.7]
R-69746	*P. elongata* 5NT	42.6	[40.1 - 45.1]	LMG 22931	R-75777	90.1	[87.8 - 92.0]
R-69746	*P. caffeinilytica* CF1T	43.3	[40.8 - 45.8]	LMG 22931	LMG 31832T	40.9	[38.4 - 43.5]

Strain 1	Strain 2	dDDH (%)	C.I. (%)	Strain 1	Strain 2	dDDH (%)	C.I. (%)
LMG 22931	R-69749	41.3	[38.8 - 43.8]	R-69608	LMG 31837^T	39.8	[37.3 - 42.3]
LMG 22931	R-69927	40.8	[38.3 - 43.4]	R-69776	*P. aspalathi* LMG 27731^T	62.8	[59.9 - 65.6]
LMG 22931	R-70006	40.9	[38.4 - 43.4]	R-69776	*P. madseniana* RP11^T	57	[54.2 - 59.7]
LMG 22931	R-70199	40.9	[38.4 - 43.5]	R-69776	*P. sediminicola* LMG 24238^T	55.4	[52.6 - 58.1]
LMG 22931	R-70211	40.8	[38.4 - 43.4]	R-69776	*P. solitsugae* 1N^T	41.2	[38.7 - 43.8]
LMG 22931	R-75471	40.9	[38.4 - 43.4]	R-69776	*P. elongata* 5N^T	42.6	[40.1 - 45.2]
LMG 22931	LMG 31837^T	39.7	[37.2 - 42.2]	R-69776	*P. caffeinilytica* CF1^T	43.7	[41.2 - 46.3]
LMG 31840	*P. aspalathi* LMG 27731^T	63	[60.1 - 65.8]	R-69776	R-75465	62.8	[59.9 - 65.6]
LMG 31840	*P. madseniana* RP11^T	57.5	[54.7 - 60.2]	R-69776	LMG 31836^T	89.5	[87.1 - 91.5]
LMG 31840	*P. sediminicola* LMG 24238^T	55.5	[52.8 - 58.3]	R-69776	R-75777	98	[97.1 - 98.6]
LMG 31840	*P. solitsugae* 1N^T	41.4	[38.9 - 43.9]	R-69776	LMG 31832^T	41.1	[38.6 - 43.7]
LMG 31840	*P. elongata* 5N^T	42.9	[40.4 - 45.5]	R-69776	R-69927	41.1	[38.6 - 43.7]
LMG 31840	*P. caffeinilytica* CF1^T	43.8	[41.2 - 46.3]	R-69776	R-70006	41.1	[38.6 - 43.7]
LMG 31840	R-69658	65.3	[62.4 - 68.1]	R-69776	R-70199	41.1	[38.6 - 43.7]
LMG 31840	R-69746	63.2	[60.3 - 66.0]	R-69776	R-70211	40.7	[38.3 - 43.3]
LMG 31840	R-75465	63.2	[60.3 - 66.0]	R-69776	R-75471	41.1	[38.6 - 43.6]
LMG 31840	LMG 31836^T	89.2	[86.8 - 91.2]	R-69776	LMG 31837^T	39.7	[37.2 - 42.2]
LMG 31840	R-69776	91.2	[89.1 - 93.0]	R-75777	*P. aspalathi* LMG 27731^T	62.8	[59.9 - 65.6]
LMG 31840	R-75777	91.6	[89.5 - 93.3]	R-75777	*P. madseniana* RP11^T	57.4	[54.6 - 60.2]
LMG 31840	LMG 31832^T	41.2	[38.7 - 43.7]	R-75777	*P. sediminicola* LMG 24238^T	55.5	[52.8 - 58.2]
LMG 31840	R-69749	43.7	[41.1 - 46.2]	R-75777	*P. solitsugae* 1N^T	41.4	[38.9 - 43.9]
LMG 31840	R-69927	41.1	[38.6 - 43.7]	R-75777	*P. elongata* 5N^T	42.8	[40.3 - 45.4]
LMG 31840	R-70006	41.1	[38.6 - 43.7]	R-75777	*P. caffeinilytica* CF1^T	43.9	[41.3 - 46.4]
LMG 31840	R-70199	41.2	[38.7 - 43.7]	LMG 31832^T	*P. aspalathi* LMG 27731^T	40.6	[38.1 - 43.1]
LMG 31840	R-70211	40.8	[38.3 - 43.3]	LMG 31832^T	*P. madseniana* RP11^T	41.9	[39.4 - 44.4]
LMG 31840	R-75471	41.1	[38.7 - 43.7]	LMG 31832^T	*P. sediminicola* LMG 24238^T	39.9	[37.5 - 42.5]
LMG 31840	LMG 31837^T	39.7	[37.2 - 42.2]	LMG 31832^T	*P. solitsugae* 1N^T	46.3	[43.7 - 48.9]
R-69608	*P. aspalathi* LMG 27731^T	63	[60.1 - 65.9]	LMG 31832^T	*P. elongata* 5N^T	43.8	[41.3 - 46.4]
R-69608	*P. madseniana* RP11^T	57.5	[54.7 - 60.2]	LMG 31832^T	*P. caffeinilytica* CF1^T	39.8	[37.3 - 42.3]
R-69608	*P. sediminicola* LMG 24238^T	55.4	[52.7 - 58.2]	LMG 31832^T	R-75777	40.9	[38.4 - 43.4]
R-69608	*P. solitsugae* 1N^T	41.4	[38.9 - 43.9]	R-69749	*P. aspalathi* LMG 27731^T	40.6	[38.1 - 43.2]
R-69608	*P. elongata* 5N^T	42.9	[40.4 - 45.5]	R-69749	*P. madseniana* RP11^T	42	[39.5 - 44.6]
R-69608	*P. caffeinilytica* CF1^T	43.9	[41.3 - 46.4]	R-69749	*P. sediminicola* LMG 24238^T	40.1	[37.6 - 42.6]
R-69608	R-69658	64.6	[61.7 - 67.4]	R-69749	*P. solitsugae* 1N^T	46.3	[43.7 - 48.9]
R-69608	R-69746	63.3	[60.4 - 66.1]	R-69749	*P. elongata* 5N^T	43.8	[41.3 - 46.4]
R-69608	R-75465	62.8	[59.9 - 65.6]	R-69749	*P. caffeinilytica* CF1^T	39.8	[37.3 - 42.3]
R-69608	LMG 31836^T	90.6	[88.3 - 92.4]	R-69749	R-75465	40.9	[38.4 - 43.5]
R-69608	LMG 31840	91.4	[89.2 - 93.1]	R-69749	LMG 31836^T	41	[38.5 - 43.5]
R-69608	R-69776	91.9	[89.8 - 93.6]	R-69749	R-69776	43.9	[41.4 - 46.5]
R-69608	R-75777	91.6	[89.5 - 93.4]	R-69749	R-75777	43.5	[41.0 - 46.1]
R-69608	LMG 31832^T	41.2	[38.7 - 43.7]	R-69749	LMG 31832^T	95.1	[93.5 - 96.3]
R-69608	R-69749	43.5	[41.0 - 46.1]	R-69749	R-69927	96.6	[95.3 - 97.5]
R-69608	R-69927	41.1	[38.6 - 43.7]	R-69749	R-70006	97	[95.8 - 97.8]
R-69608	R-70006	41.1	[38.6 - 43.7]	R-69749	R-70199	97.6	[96.6 - 98.3]
R-69608	R-70199	41.2	[38.7 - 43.7]	R-69749	R-70211	97.5	[96.4 - 98.2]
R-69608	R-70211	40.9	[38.4 - 43.4]	R-69749	R-75471	97.5	[96.5 - 98.3]
R-69608	R-75471	41.1	[38.6 - 43.7]	R-69749	LMG 31837^T	40.8	[38.3 - 43.3]

Strain 1	Strain 2	dDDH (%)	C.I. (%)
R-69927	*P. aspalathi* LMG 27731^{T}	40.6	[38.1 - 43.1]
R-69927	*P. madseniana* RP11^{T}	41.9	[39.4 - 44.4]
R-69927	*P. sediminicola* LMG 24238^{T}	40	[37.5 - 42.5]
R-69927	*P. solitsugae* 1N^{T}	46.4	[43.8 - 49.0]
R-69927	*P. elongata* 5N^{T}	43.7	[41.2 - 46.3]
R-69927	*P. caffeinilytica* CF1^{T}	39.7	[37.2 - 42.2]
R-69927	R-75465	40.7	[38.2 - 43.3]
R-69927	LMG 31836^{T}	40.6	[38.1 - 43.1]
R-69927	R-75777	40.9	[38.4 - 43.4]
R-69927	LMG 31832^{T}	95.2	[93.7 - 96.4]
R-69927	R-70006	97.1	[95.9 - 97.9]
R-69927	R-70199	97.8	[96.9 - 98.5]
R-69927	R-70211	97.8	[96.9 - 98.5]
R-69927	R-75471	98.1	[97.3 - 98.7]
R-70006	*P. aspalathi* LMG 27731^{T}	40.5	[38.1 - 43.1]
R-70006	*P. madseniana* RP11^{T}	41.9	[39.4 - 44.5]
R-70006	*P. sediminicola* LMG 24238^{T}	40	[37.5 - 42.5]
R-70006	*P. solitsugae* 1N^{T}	46.4	[43.8 - 49.0]
R-70006	*P. elongata* 5N^{T}	43.7	[41.2 - 46.3]
R-70006	*P. caffeinilytica* CF1^{T}	39.7	[37.2 - 42.3]
R-70006	R-75465	40.7	[38.2 - 43.2]
R-70006	LMG 31836^{T}	40.6	[38.1 - 43.1]
R-70006	R-75777	40.9	[38.4 - 43.4]
R-70006	LMG 31832^{T}	96.1	[94.7 - 97.1]
R-70006	R-70199	98.1	[97.2 - 98.7]
R-70006	R-70211	97.7	[96.8 - 98.4]
R-70006	R-75471	98.4	[97.6 - 98.9]
R-70199	*P. aspalathi* LMG 27731^{T}	40.6	[38.1 - 43.1]
R-70199	*P. madseniana* RP11^{T}	41.9	[39.4 - 44.5]
R-70199	*P. sediminicola* LMG 24238^{T}	39.9	[37.4 - 42.5]
R-70199	*P. solitsugae* 1N^{T}	46.4	[43.8 - 48.9]
R-70199	*P. elongata* 5N^{T}	43.8	[41.2 - 46.3]
R-70199	*P. caffeinilytica* CF1^{T}	39.8	[37.3 - 42.3]
R-70199	R-75465	40.7	[38.2 - 43.3]
R-70199	LMG 31836^{T}	40.7	[38.2 - 43.2]
R-70199	R-75777	40.9	[38.4 - 43.4]
R-70199	LMG 31832^{T}	96.2	[94.9 - 97.2]
R-70199	R-70211	98.4	[97.6 - 98.9]
R-70199	R-75471	98.5	[97.8 - 99.0]
R-70211	*P. aspalathi* LMG 27731^{T}	40.7	[38.2 - 43.2]
R-70211	*P. madseniana* RP11^{T}	41.9	[39.4 - 44.5]
R-70211	*P. sediminicola* LMG 24238^{T}	40	[37.5 - 42.5]
R-70211	*P. solitsugae* 1N^{T}	46.4	[43.9 - 49.0]
R-70211	*P. elongata* 5N^{T}	43.8	[41.3 - 46.4]
R-70211	*P. caffeinilytica* CF1^{T}	39.8	[37.3 - 42.3]
R-70211	R-75465	40.6	[38.1 - 43.2]
R-70211	LMG 31836^{T}	40.7	[38.2 - 43.2]
R-70211	R-75777	40.8	[38.3 - 43.4]
R-70211	LMG 31832^{T}	96.1	[94.7 - 97.1]
R-70211	R-75471	98.5	[97.8 - 99.0]
R-75471	*P. aspalathi* LMG 27731^{T}	40.5	[38.0 - 43.0]
R-75471	*P. madseniana* RP11T	41.9	[39.4 - 44.4]
R-75471	*P. sediminicola* LMG 24238^{T}	40	[37.5 - 42.5]
R-75471	*P. solitsugae* 1N^{T}	46.5	[43.9 - 49.1]
R-75471	*P. elongata* 5N^{T}	43.8	[41.2 - 46.3]
R-75471	*P. caffeinilytica* CF1^{T}	39.8	[37.3 - 42.3]
R-75471	R-75777	40.9	[38.4 - 43.4]
R-75471	LMG 31832^{T}	96.6	[95.3 - 97.5]
LMG 31837^{T}	*P. aspalathi* LMG 27731^{T}	39.3	[36.8 - 41.8]
LMG 31837^{T}	*P. madseniana* RP11^{T}	41	[38.5 - 43.5]
LMG 31837^{T}	*P. sediminicola* LMG 24238^{T}	38.9	[36.4 - 41.4]
LMG 31837^{T}	*P. solitsugae* 1N^{T}	41	[38.5 - 43.5]
LMG 31837^{T}	*P. elongata* 5N^{T}	44.8	[42.2 - 47.3]
LMG 31837^{T}	*P. caffeinilytica* CF1^{T}	39.1	[36.6 - 41.6]
LMG 31837^{T}	R-75465	39.3	[36.8 - 41.8]
LMG 31837^{T}	LMG 31836^{T}	39.5	[37.0 - 42.1]
LMG 31837^{T}	R-75777	39.8	[37.3 - 42.3]
LMG 31837^{T}	LMG 31832^{T}	40.7	[38.2 - 43.2]
LMG 31837^{T}	R-69927	40.6	[38.1 - 43.1]
LMG 31837^{T}	R-70006	40.6	[38.1 - 43.1]
LMG 31837^{T}	R-70199	40.5	[38.0 - 43.0]
LMG 31837^{T}	R-70211	40.4	[37.9 - 43.0]
LMG 31837^{T}	R-75471	40.5	[38.1 - 43.1]
